# BRD4 promotes endodermal cell fate during mammalian lung development

**DOI:** 10.1172/jci.insight.194683

**Published:** 2026-02-03

**Authors:** Hongbo Wen, Derek C. Liberti, Prashant Chandrasekaran, Shahana Parveen, Kwaku K. Quansah, Mijeong Kim, Ana N. Lange, Abigail T. Marquis, Sylvia N. Michki, Annabelle Jin, MinQi Lu, Ayomikun A. Fasan, Sriyaa Suresh, Shawyon P. Shirazi, Lisa R. Young, Jennifer M.S. Sucre, Maria C. Basil, Rajan Jain, David B. Frank

**Affiliations:** 1 Department of Pediatrics, Division of Cardiology, Perelman School of Medicine, University of Pennsylvania, and; 2 CHOP Cardiovascular Institute, Children’s Hospital of Philadelphia, Philadelphia, Pennsylvania, USA.; 3 Penn Cardiovascular Institute,; 4 Penn-CHOP Lung Biology Institute, and; 5 Department of Medicine, Perelman School of Medicine, University of Pennsylvania, Philadelphia, Pennsylvania, USA.; 6 Department of Pediatrics, Division of Pulmonary and Sleep Medicine, Perelman School of Medicine, University of Pennsylvania, Children’s Hospital of Philadelphia, Pennsylvania, USA.; 7 Department of Pediatrics and Cell & Developmental Biology, Vanderbilt University, Nashville, Tennessee, USA.; 8 Department of Medicine and Cell & Developmental Biology, Perelman School of Medicine, University of Pennsylvania, Philadelphia, Pennsylvania, USA.

**Keywords:** Development, Pulmonology, Embryonic development, Mouse models, Mouse stem cells

## Abstract

Lung development relies on diverse cell intrinsic and extrinsic mechanisms to ensure proper cellular differentiation and compartmentalization. In addition, it requires precise integration of multiple signaling pathways to temporally regulate morphogenesis and appropriate cell specification. To accomplish this, organogenesis relies on epigenetic and transcriptional regulators to promote cell fate and inhibit alternative cell fates. Using genetic mouse and human embryonic stem cell (hESC) differentiation models, tissue explants, and single-cell transcriptomic analysis, we demonstrated that Bromodomain Containing Protein 4 (BRD4) is required for mammalian lung morphogenesis and cell fate. Endodermal deletion of BRD4 impaired epithelial-mesenchymal crosstalk, leading to disrupted proximal-distal patterning and branching morphogenesis. Moreover, temporal deletion of BRD4 revealed developmental stage–specific defects in airway and alveolar epithelial cell specification with a predominant role in proximal airway cell fate. Similarly, BRD4 promoted lung endodermal cell differentiation into airway lineages in a hESC-derived lung organoid model. Together, these data demonstrate that BRD4 orchestrates early lung morphogenesis and separately regulates cell specification, indicating a multifunctional and evolutionarily conserved role for BRD4 in mammalian lung development.

## Abstract 2

The transcriptional regulator, BRD4, regulates gene expression and cell differentiation of lung endodermal progenitors into epithelial cells of the lung airways.

## Graphical abstract

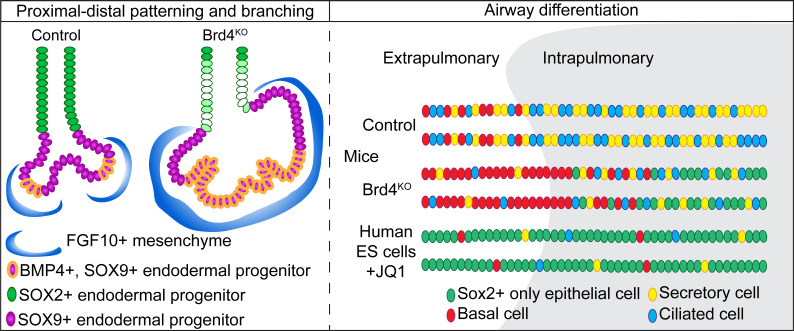

## Introduction

Lung development relies on precise, highly stereotyped intercellular communication networks to establish the complex architecture of the adult organ. In particular, epithelial-mesenchymal signaling guides branching morphogenesis via well-investigated molecular pathways, including SHH, FGF, BMP, retinoic acid, TGF-β, and WNT (1–3). More recently, studies have demonstrated that lung morphogenesis relies on epigenetic factors to ensure the proper epithelial lineage specification, proliferation, and maturation necessary to generate a functional lung (4–17). Epithelial cell fate decisions occur in a spatially specific manner and are major drivers of not only lung development but also adult regeneration after injury (18–20). The ability of epigenetic factors to manipulate epithelial cell fate has made them attractive targets for amelioration of human disease, but our understanding of these factors remains limited (21, 22). Elucidating the role of epigenetic factors in epithelial cell fate is essential to develop targeted therapies to improve lung disease outcomes.

Bromodomain containing protein 4 (BRD4) is an epigenetic factor with multiple roles in transcription regulation. BRD4 has been shown to recognize and bind acetylated lysine residues on histones to serve as a scaffold to recruit transcription factors, act as a kinase and histone acetyltransferase, and maintain chromatin architecture (23–26). Due to its broad functionality, BRD4 is essential for early embryonic mouse development and pluripotent stem cell identity maintenance (27, 28). In addition, BRD4 is a key regulator of normal cell cycle progression (29, 30). BRD4 has been implicated in the promotion of pulmonary arterial hypertension and pulmonary fibrosis, but the role of this factor during lung development is largely unknown (31, 32).

Using in vivo genetic mouse models, ex vivo lung explant assays, single-cell transcriptomics, and human embryonic stem cell–derived (hESC-derived) lung endoderm organoids, we investigated the role of BRD4 in the developing lung endoderm. We show that endodermal deletion of BRD4 results in the emergence of cystic distal airway structures and perinatal lethality due to respiratory failure. Furthermore, we demonstrate that endodermal BRD4-deficient mice exhibit impaired epithelial-mesenchymal crosstalk, lung branching morphogenesis, proximal-distal patterning, and cell specification. Temporal deletion of BRD4 in lung endoderm reveals a separate role in cell specification with a predominant effect on proximal airway cell fate, a function observed in both mouse and human cells. Taken together, our data demonstrate that BRD4 orchestrates lung morphogenesis and proper epithelial lineage determination.

## Results

### Endodermal BRD4 is required for lung morphogenesis and perinatal survival.

**Figure 1:**
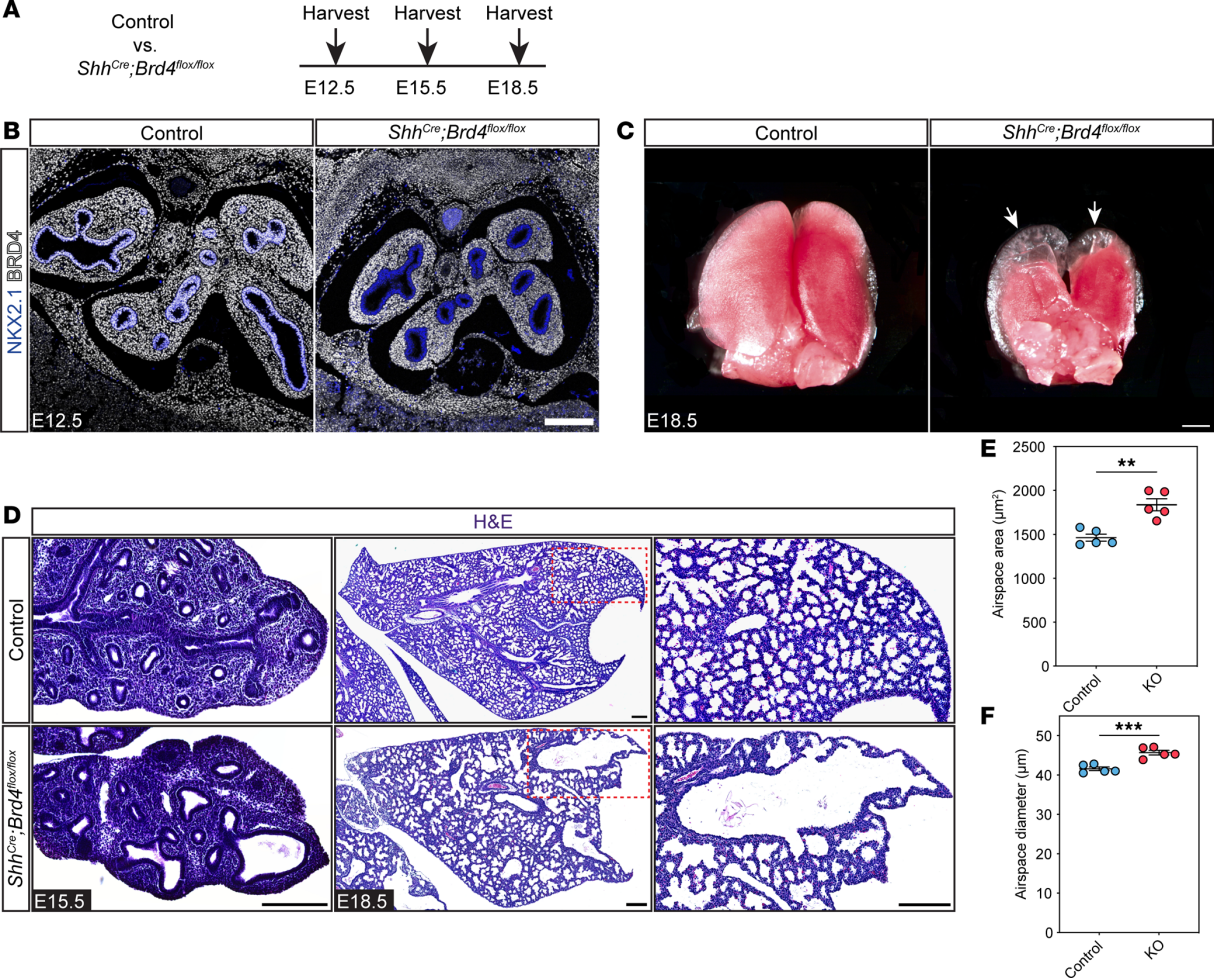
Endodermal BRD4 is required for lung morphogenesis and perinatal survival. (**A**) Experimental schematic indicating the time points analyzed. (**B**) IHC for NKX2.1 and BRD4 at E12.5. Scale bar: 200 μm. (**C**) Bright-field images of whole control and mutant lungs at E18.5. White arrows mark cystic distal airway structures. Scale bar: 2 mm. (**D**) H&E staining at E15.5 and E18.5. Scale bars: 200 μm. (**E** and **F**) Quantification of morphological changes shown in **D** at E18.5. Data are represented as mean ± SEM. Two-tailed *t* test: **P ≤ 0.01, ***P ≤ 0.001, *n* = 5.

To determine the role of BRD4 in the developing lung, we generated *Shh^Cre^* *Brd4^fl/fl^* mice, allowing conditional deletion of BRD4 in the anterior foregut endoderm during lung development from E9.5 onward (Figure 1A) (25, 33). BRD4 is nearly ubiquitously expressed in all lung cell types including those of the epithelial lineages (Supplemental Figure 1, A and B; supplemental material available online with this article; https://doi.org/10.1172/jci.insight.194683DS1). While we detected BRD4 protein throughout the developing embryo, we confirmed complete loss of BRD4 from the lung endoderm in *Shh^Cre^* *Brd4^fl/fl^* mice at E12.5 (Figure 1B). BRD4-deficient lungs exhibited abnormal morphogenesis, including the formation of cystic distal airway structures across development (Figure 1, C and D). Additionally, morphological analysis of tissue sections at E18.5 revealed statistically significant expansion of all other distal airspaces in mutant lungs compared with controls (Figure 1, D–F). *Shh^Cre^* *Brd4^fl/fl^* mice did not survive until weaning, and we found that *Shh^Cre^* *Brd4^fl/fl^* mice became cyanotic and perished shortly after birth, consistent with respiratory failure (Supplemental Figure 1, C and D). After harvest and placement in formalin, mutant lungs floated with a large collection of large air bubbles in their cystic airway structures, suggesting that *Shh^Cre^* *Brd4^fl/fl^* mice could inhale with an inability to appropriately exhale air out (Supplemental Figure 1E).

### Loss of endodermal BRD4 disrupts epithelial-mesenchymal crosstalk.

**Figure 2:**
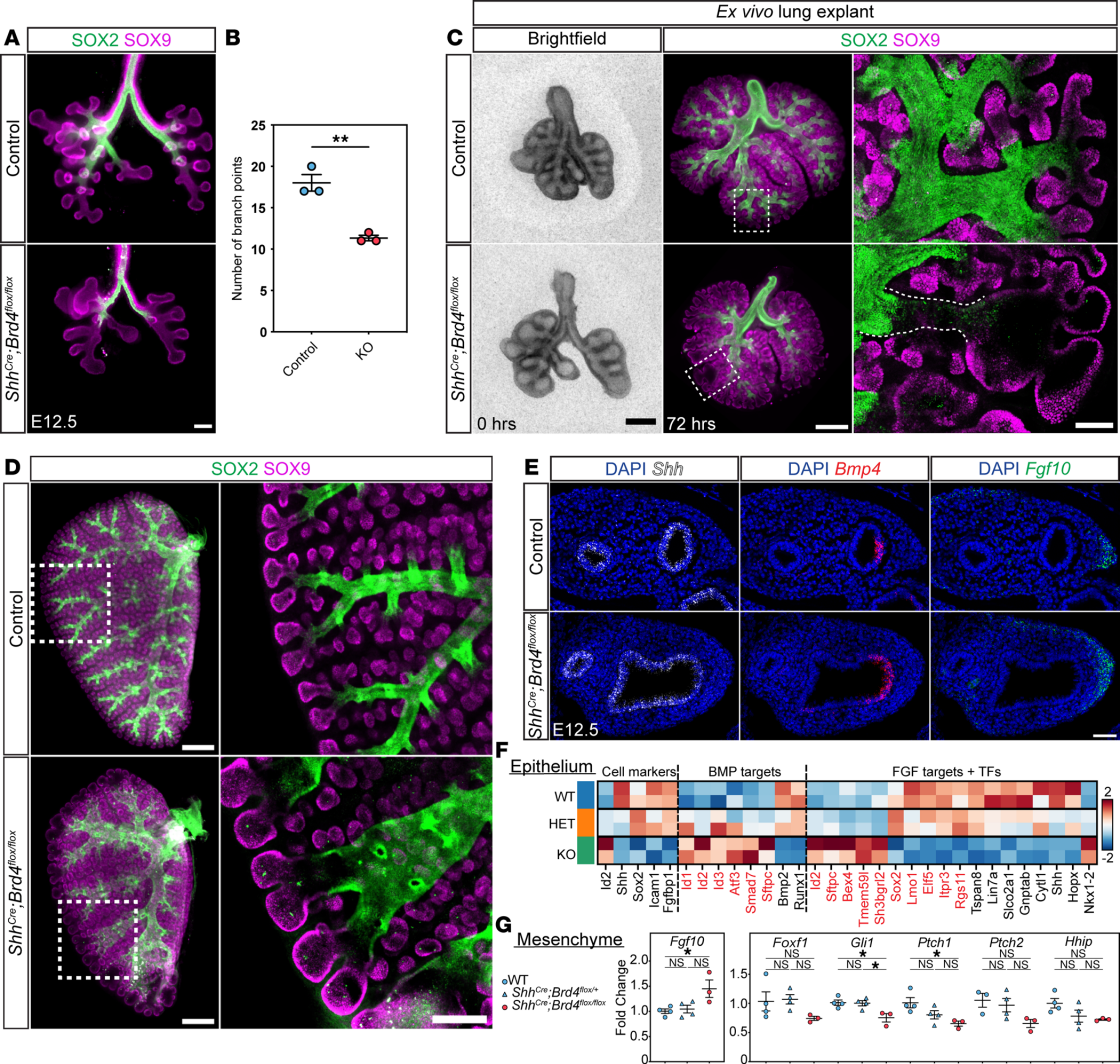
Loss of endodermal BRD4 disrupts epithelial-mesenchymal crosstalk. (**A** and **B**) Wholemount IHC for SOX2 and SOX9 to assess branching at E12.5. Scale bar: 200 μm. Quantification data are represented as mean ± SEM. Two-tailed *t* test: **P ≤ 0.01, *n* = 3. (**C**) Left: bright-field images of control and mutant lungs at E12.5. Scale bar: 500 μm. Middle: wholemount IHC for SOX2 and SOX9 after 3 days of culture ex vivo. Scale bar: 500 μm. Dashed white boxes indicate branching tips in control and mutant lungs. Right: magnified images of branching tips. Dashed white lines outline a distal airway. Scale bar: 100 μm. (**D**) Wholemount IHC for SOX2 and SOX9 at E15.5. Scale bar: 500 μm. Dashed white boxes mark magnified areas shown in the right panels. Scale bar: 100 μm. (**E**) RNA FISH for *Shh*, *Bmp4*, and *Fgf10* in control and mutant E12.5 lungs. Scale bar: 50 μm. (**F**) Matrix plots of differentially expressed genes by RNA-seq of E12.5 epithelium including cell markers, BMP signaling targets, and FGF signaling targets. Genes in red for BMP and FGF targets are positively correlated with activation of each respective signaling pathway. (**G**) Gene expression analysis by qPCR for *Fgf10* (left box) and SHH target genes in E12.5 mesenchyme. Quantification data are represented as mean ± SEM. One-way ANOVA: *P ≤ 0.05, *n* = 3–4.

To assess how loss of BRD4 results in the formation of cystic distal airway structures, we investigated early branching morphogenesis in control and mutant (*Shh^Cre^* *Brd4^fl/fl^*) lungs. As early as E12.5, we observed impaired branching in BRD4-deficient mice (Figure 2, A and B). To determine whether the observed defect was lung intrinsic, we harvested control and mutant lungs at E12.5 and cultured them ex vivo for 3 days (Figure 2C). Mutant lungs exhibited fewer and dilated airways with diminished SOX2 expression in distal airways in addition to the formation of cystic distal airway structures, consistent with our in vivo observations (Figure 2, C and D). We did not observe any significant differences in proliferation of proximal or distal epithelial cells or cell death in control or mutant mice (Supplemental Figure 2, A–C).

To determine whether abnormal branching may be a result of altered epithelial-mesenchymal communication, we investigated the expression of critical branching factors *Shh*, *Bmp4*, and *Fgf10* using multiplexed RNA FISH at E12.5 and E15.5 (Figure 2E and Supplemental Figure 2D). The SHH/BMP4/FGF10 signaling axis has been well characterized in the lung with feedback loops between SHH and FGF signaling (34–37). *Shh* is typically expressed throughout the developing lung endoderm (Figure 2E and Supplemental Figure 2D). However, in BRD4-deficient mice, *Shh* expression was mildly diminished at the distal tips of cystic distal airway structures (Figure 2E), which was more prominent at E15.5 (Supplemental Figure 2D). Moreover, while *Bmp4* expression was restricted to the center of the distal tips of airway structures in control lungs, *Bmp4* expression expanded throughout the distal tip in BRD4-deficient mice at both time points (Figure 2E and Supplemental Figure 2D). We confirmed these changes using qPCR on EPCAM^+^ cells isolated by FACS at E15.5 and observed a significant decrease in *Shh* and increase in *Bmp4* (Supplemental Figure 2E). Additionally, *Fgf10* expression was increased in BRD4 mutant lungs at E12.5 and E15.5. Similar findings were observed in a recent study in lung development, revealing that FGF signaling components including ETV5 mediate the FGF-SHH signaling loop (35). Thus, we performed FISH for *Etv5* and *Fgfr2* but observed no changes in epithelial expression between controls and mutants at E15.5 (Supplemental Figure 2F).

To further explore dysregulation of SHH/BMP4/FGF10 pathways involved in epithelial-mesenchymal crosstalk, we used FACS of epithelial and mesenchymal-enriched cells from 3 different litters of E12.5 Cre negative (WT), heterozygous, and homozygous BRD4 mutant mice. Subsequently, we performed RNA-seq on the epithelial cells and qPCR on isolated mesenchymal cells. Of 15,575 genes detected, only 6 genes were differentially expressed between Cre negative WT and *Shh^Cre^* *Brd4^fl/+^* embryonic lung epithelial cells including *Shh*, *Id1*, *Rnu1b2*, *Comtd1*, *Rnps1*, and *Atf6b*, indicating that *Shh* haploinsufficiency using *Shh^Cre^* does not result in significant gene dysregulation in BRD4 mutants (Supplemental Tables 1–3). Assessment of gene regulation revealed that BRD4 mutants exhibited significant dysregulation of multiple genes important in lung and stem cell development (Supplemental Figure 3, A and B). Moreover, we confirmed a decrease in *Sox2* and the recently described transitional zone of differentiation cells expressing both *Icam1* and *Fgfbp1* (Figure 2F) but no change in *Sox9* (38). Further differential gene expression analysis confirmed decreased expression of multiple target genes of BMP signaling in the epithelium (Figure 2F). While we observed an increase in *Fgf10* expression in the mesenchyme (Figure 2G), expression of a previously published panel of FGF10 target genes in the epithelium and mesenchyme was inconsistent, with only half of the statistically significant target gene positively correlated with increased *Fgf10* (Figure 2F) (39). However, qPCR of SHH target genes in the mesenchyme did reveal trends and statistically significant decreased expression of multiple SHH signaling transcriptional targets including *Gli1* and *Ptch1* (Figure 2G). Together, our data show that loss of BRD4 leads to impaired epithelial-mesenchymal crosstalk, in part, through dysregulation of SHH and BMP signaling.

### SHH activation partially rescues BRD4 mutant cystic distal airway structure phenotype.

To further characterize the potential mechanism of SHH signaling in BRD4 mutants, we activated SHH signaling with Purmorphamine (PMA) in our ex vivo lung explants (40). Treatment of mutant explants with PMA over 72 hours partially rescued the phenotype (Supplemental Figure 3, C and D). Quantification of SOX2 expression revealed a statistically significant reemergence of SOX2 expression and a modest reduction of the distal airway structures in the mutant lung with PMA treatment (Supplemental Figure 3, E and F). However, morphometric analysis revealed a reduced tip number in both the control group and mutant group with PMA treatment (Supplemental Figure 3G).

### Loss of endodermal BRD4 results in impaired epithelial cell differentiation in large distal cysts.

**Figure 3:**
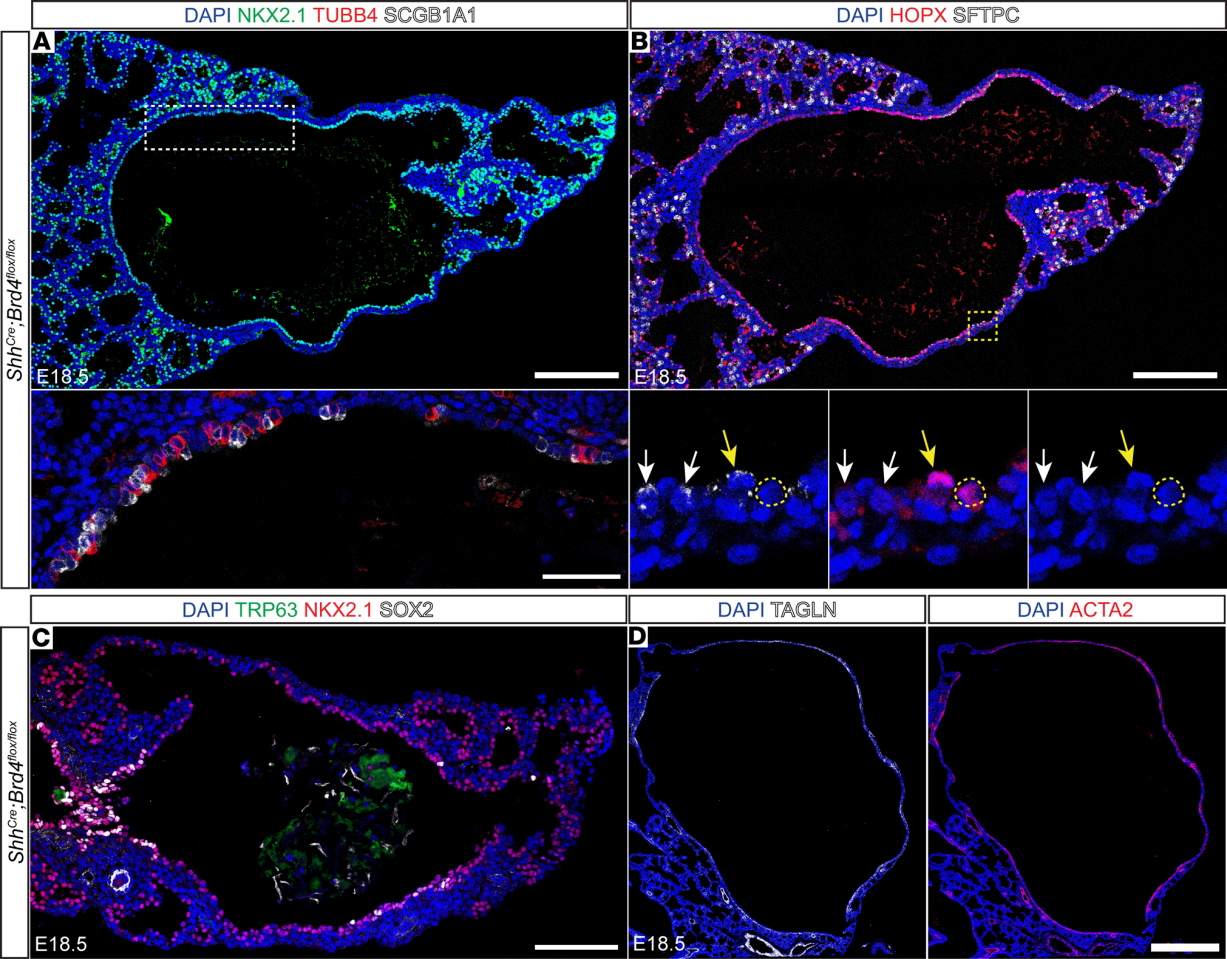
Loss of endodermal BRD4 results in impaired epithelial cell differentiation in large distal cysts. (**A**) IHC for NKX2.1, TUBB4, and SCGB1A1 in BRD4 mutant E18.5 lungs. Scale bar: 200 μm. Dashed white box marks magnified region shown in the lower image. Scale bar: 50 μm. (**B**) IHC for HOPX and SFTPC in BRD4 mutant E18.5 lungs. Scale bar: 200 μm. Dashed yellow box marks magnified region shown in the lower image. Dashed yellow circles mark AT1 cells, white arrows mark AT2 cells, and yellow arrows mark HOPX^+^SFTPC^+^ cells. (**C**) IHC for basal cell marker, TRP63, NKX2.1, and SOX2 in BRD4 mutant lung cystic distal airway structures at E18.5. Scale bar: 150 μm. (**D**) IHC for TAGLN and ACTA2 in BRD4 mutant lung cystic distal airway structures at E18.5. Scale bar: 500 μm.

To determine how early patterning defects in BRD4 mutants affect lung maturation, we examined the composition of the cystic distal airway structures that exist only in BRD4 mutant lungs. The apical surfaces of these structures were lined with cuboidal epithelial cells and were associated with deficits in appropriate differentiation of proximal airway and distal alveolar epithelial cells (Figure 3, A and B). Epithelial cells in the proximal portion of cystic distal airway structures weakly expressed markers for secretory and multiciliated cells, including SCGB1A1 and TUBB4 (Figure 3A), while cuboidal epithelial cells in the distal portion expressed alveolar epithelial type 1 (AT1) and AT2 markers, including HOPX and SFTPC (Figure 3B). However, HOPX expression expanded into the proximal region of the cystic distal airway structures, and some SFTPC^+^ cells also expressed HOPX (Figure 3B). Interestingly, no basal cells were noted within the large distal cysts (Figure 3C). In addition to compromised lineage restriction and maturation, these cystic distal airway structures were encased in ectopic smooth muscle, suggesting impaired epithelial-mesenchymal crosstalk (Figure 3D).

### Single-cell RNA-seq indicates global endodermal differentiation defects in BRD4 mutants.

**Figure 4:**
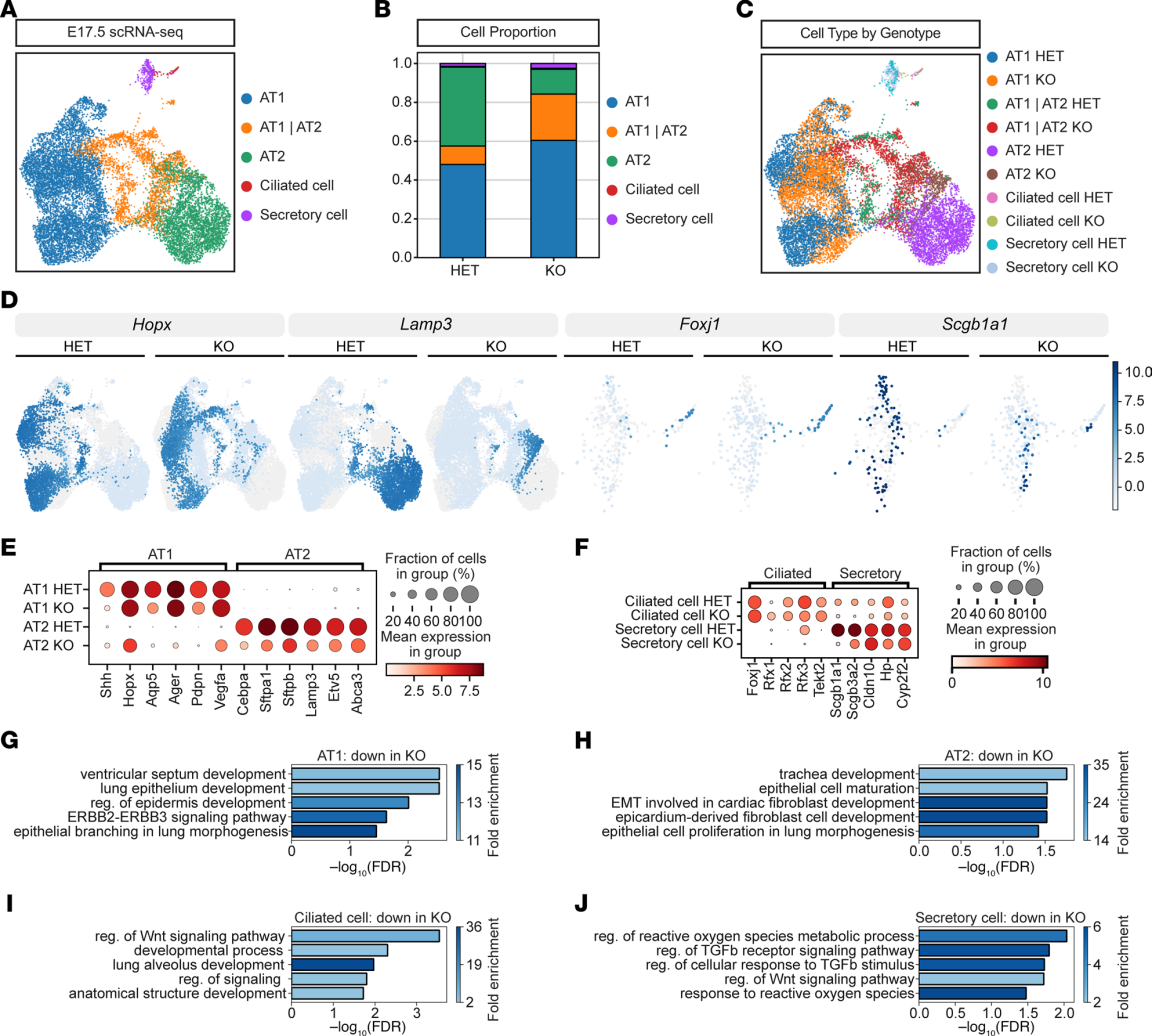
scRNA-seq indicates global endodermal differentiation defects in BRD4 mutants. (**A**) UMAP embedding of BRD4 heterozygous and homozygous KO lung epithelial cells colored by cell type. (**B**) Cell compositional changes in BRD4 heterozygous and homozygous knockout lungs based on cell proportion. (**C**) UMAP embedding of epithelial cells colored by genotype and cell type. (**D**) UMAPs embedding of epithelial cells colored by cell marker genes. UMAPs for *Foxj1* and *Scgb1a1* are a magnification of the proximal airway cell clusters. (**E**) Dot plots of expression of AT1 and AT2 cell marker genes. Dot size indicates the proportion of cells within a cluster expressing a gene, and color intensity indicates the relative expression level. (**F**) Dot plots of expression of ciliated and secretory cell marker genes. (**G**–**J**) Gene ontology (GO) analysis for top 5 ranked categories using downregulated (down) gene enrichment sets in BRD4 homozygous embryonic lungs. GO analysis for AT1 cells (**G**), AT2 cells (**H**), ciliated cells (**I**), and secretory cells (**J**).

To explore how loss of BRD4 affects epithelial cell differentiation and transcription globally, we FACS-isolated heterozygous control and homozygous BRD4-deficient EPCAM^+^ cells at E17.5 and performed single-cell RNA-seq. ScRNA-seq) using the 10X Genomics Chromium droplet-based scRNA-seq platform. After quality control, we sequenced over 6,520 heterozygous control and 7,151 homozygous KO cells. Cell clustering and cell annotation confirmed the presence of major intrapulmonary epithelial cell populations in the lung, including AT1, AT2, secretory, and multiciliated airway cells (Figure 4A). Total cell number and cell proportion of epithelium differed between control and BRD4 mutant mice (Figure 4B and Supplemental Figure 4A). Cell cycle phase analysis of our scRNA-seq data revealed very mild differences in the number or proportion of each phase for each cell type, indicating little change in proliferation (Supplemental Figure 4B). UMAP embedding by genotype and cell type showed predominantly segregated cell clustering, indicating significant transcriptome changes with loss of BRD4 (Figure 4, C and D). These transcriptomic changes were represented by both upregulated and downregulated genes (Supplemental Figure 4, C–F) and significant canonical cell marker gene dysregulation (Figure 4, E and F). In BRD4 mutant AT2s, there was a loss of AT2 cell markers concomitant with a gain in AT1 cell markers (Figure 4E). In addition, profiling marker gene expression in control and mutant samples revealed an attenuation of mature marker gene expression in secretory cells (Figure 4F).

To determine additional mediators of our cell state changes, we performed overrepresentation analyses (ORA) on the transcriptomes of control and BRD4 mutant AT1s, AT2s, ciliated cells, and secretory cells. ORA of AT1s, AT2s, ciliated cells, and secretory cells identified downregulation of genes associated with lung epithelial development and important pathways such as WNT and TGF-β signaling (Figure 4, G–J). We also assessed multiple signaling pathways and stress responses using Hallmark scoring on gene set enrichment analysis in both proximal and distal airway lineages and observed significant dysregulation of cell morphogenesis, inflammatory responses, and multiple signaling pathways (Supplemental Figure 4G).

### BRD4 is required for proximal and distal airway epithelial cell differentiation and maturation.

**Figure 5:**
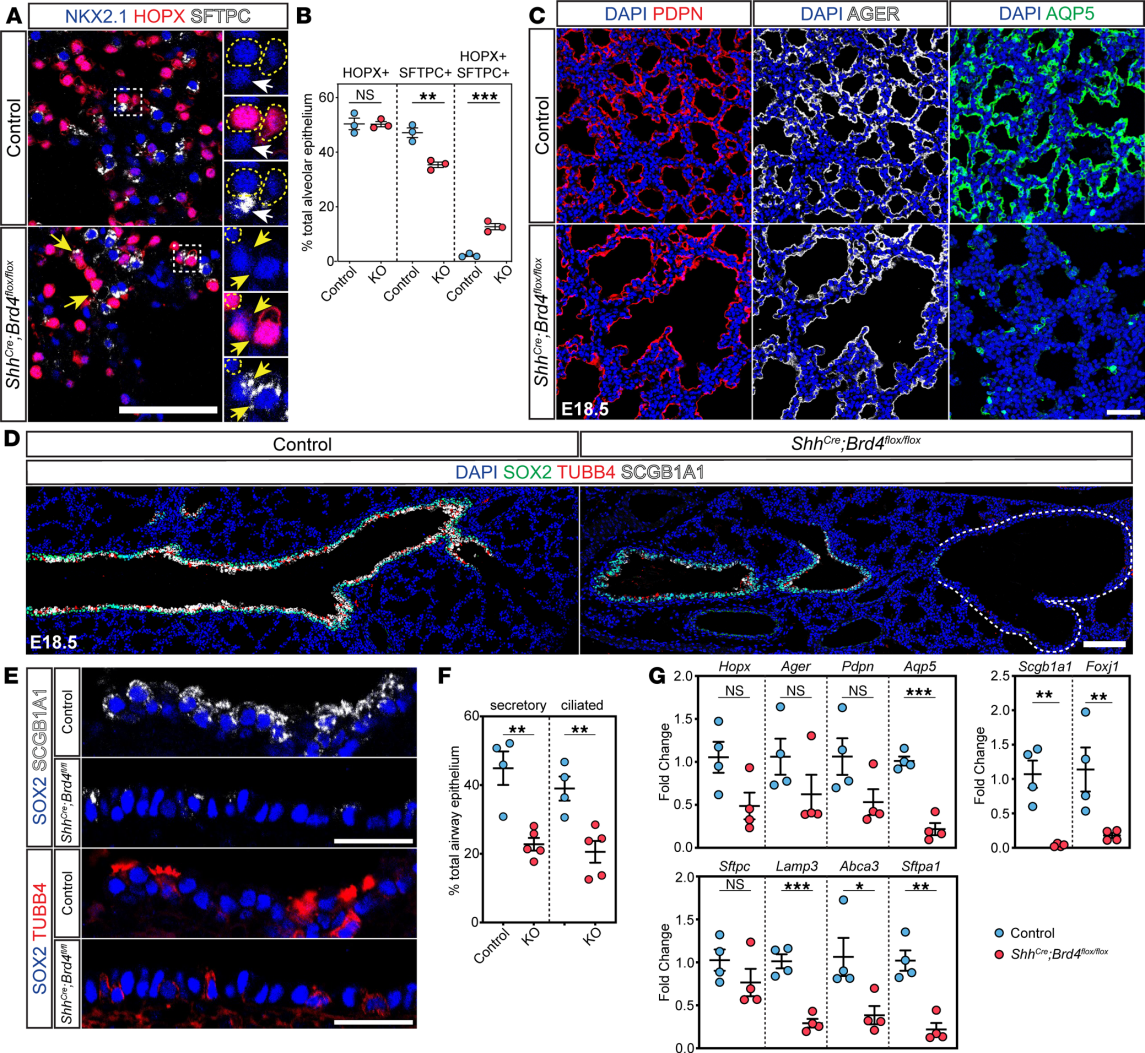
BRD4 is required for proximal and distal airway epithelial cell differentiation and maturation. (**A**) IHC for NKX2.1, HOPX, and SFTPC in control and mutant E18.5 lungs. Dashed yellow circles mark AT1 cells, white arrows mark AT2 cells, and yellow arrows mark HOPX^+^SFTPC^+^ cells. Scale bar: 50 μm. (**B**) Quantification of AT1, AT2, and HOPX^+^SFTPC^+^ cells at E18.5 in control versus mutant lungs as shown in **A**. Quantification data are represented as mean ± SEM. Two-tailed *t* tests: **P ≤ 0.01, ***P ≤ 0.001, *n* = 3. (**C**) IHC for PDPN, AGER, and AQP5 in control and mutant E18.5 lungs. Scale bar: 50 μm. (**D**) IHC for SOX2, TUBB4, and SCGB1A1 in control and mutant E18.5 lungs. Scale bar: 100 μm. Dashed white line outlines a cystic distal airway structure. (**E**) IHC for SOX2, TUBB4, and SCGB1A1 in magnified airway regions of control and BRD4 mutant E18.5 lungs. For BRD4 mutants, images reflect airways terminating into cystic distal airway structures. Scale bar: 25 μm. (**F**) Quantification of the percentages of secretory (left) and ciliated (right) cells in the airway regions. Quantification data are represented as mean ± SEM. Two-tailed *t* tests: **P ≤ 0.01, *n* ≥ 4. (**G**) qPCR analysis of AT1 marker genes (top left), AT2 marker genes (bottom left), and airway marker genes (right) in control (blue) and mutant (red) E18.5 lung epithelium. Quantification data are represented as mean ± SEM. Two-tailed t test: *P ≤ 0.05, **P ≤ 0.01, ***P ≤ 0.001, *n* = 4.

To confirm our bioinformatic findings of impaired alveolar epithelial cell differentiation, we performed histological analysis on E18.5 control and BRD4 mutant lungs. Consistent with loss of AT2 cell capture in our scRNA-seq data set, we observed a decrease in the percentage of AT2 cells (Figure 5, A and B). Moreover, we observed an increase in cells expressing both SFTPC and HOPX, suggesting impaired AT2 cell fate specification. These SFTPC^+^HOPX^+^ cells in the alveolar compartment did not express the more mature AT2 cell marker LAMP3, but rare exceptions of LAMP3^+^HOPX^+^ cells were present in cystic distal airway structures (Supplemental Figure 5A). Additionally, we observed no proliferation or cell death changes in AT1s, AT2s, or AT1/AT2s (Supplemental Figure 5, B and C), consistent with previously published data revealing a very low level of alveolar epithelial proliferation at E18.5 (18, 41). BRD4-deficient AT1 cells also failed to fully mature. While they expressed PDPN and AGER in the alveolar compartment and within the cystic distal airway structures, BRD4-deficient AT1 cells exhibited minimal expression of AQP5, a marker observed later in AT1 differentiation (Figure 5C and Supplemental Figure 5D) (41). Despite a lack of maturation of AT1 cells that communicate with the capillary bed, capillary endothelial development appeared mostly unaffected in BRD4 mutant lungs (Supplemental Figure 5, E–H).

Although BRD4 mutant airways often transitioned abruptly into cystic distal airway structures, some structures appeared to terminate into relatively normal distal saccules, allowing for continued formation of the future alveolar compartment (Figure 5D). While secretory cells expressed SCGB1A1 throughout the proximal airways in controls, there was a marked decrease in SCGB1A1 protein in the BRD4 mutant airways (Figure 5E). Similarly, multiciliated cells appeared to lose TUBB4 marker gene expression in BRD4 mutant airways (Figure 5E). We performed quantification of the number of secretory and multiciliated cells in the proximal airway compartments and found approximately a 50% reduction in both cell lineages (Figure 5F). To corroborate these deficits in lung endodermal cell fate, we performed qPCR analysis of control and mutant lung epithelial cells (Figure 5G). Similar to protein expression studies, we noted similar downregulation of lung epithelial cell fate marker genes.

**Figure 6:**
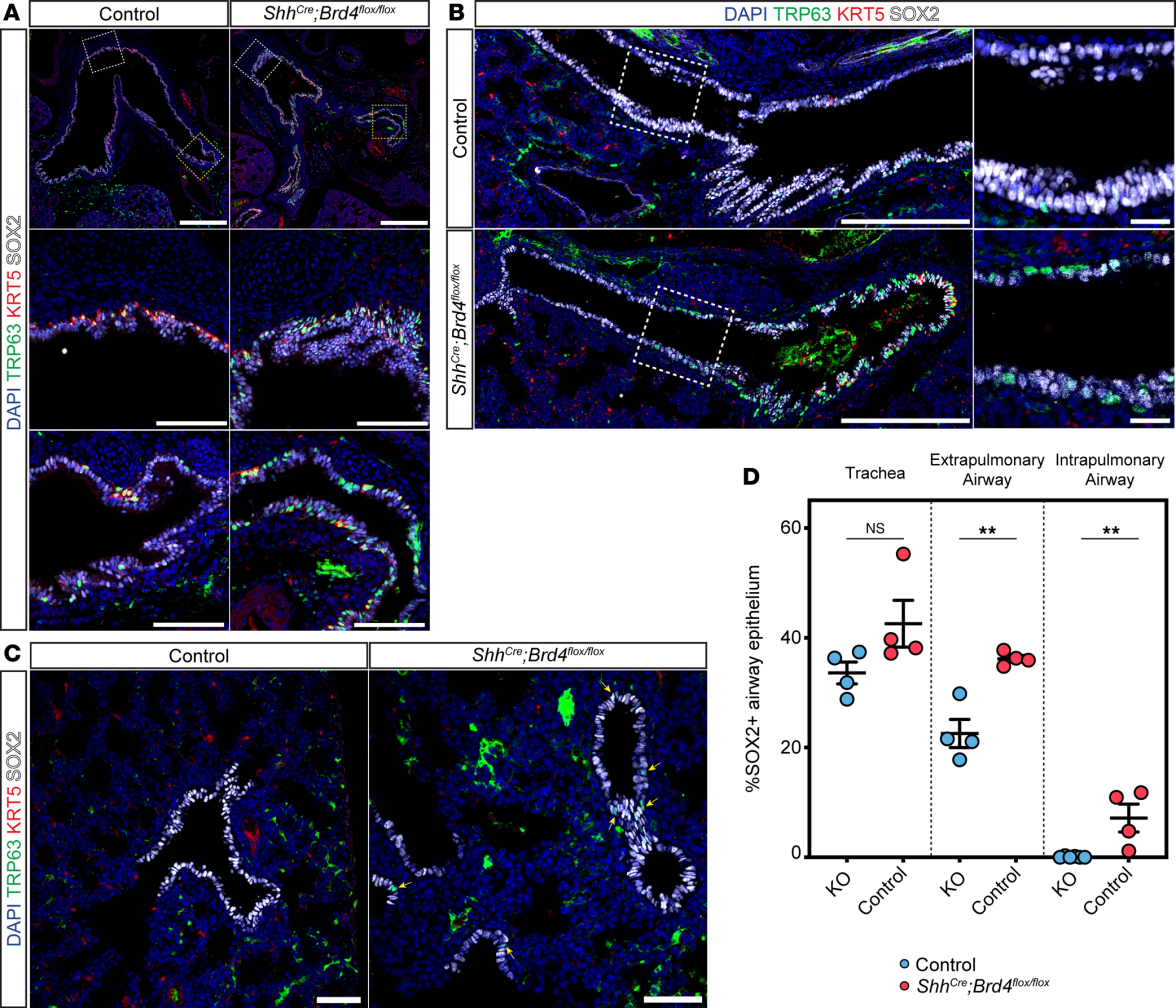
BRD4 promotes lung basal cell differentiation in extra- and intrapulmonary proximal airways. (**A**) IHC for TRP63, KRT5, and SOX2 in control and BRD4 mutant E18.5 trachea (top and middle panels) and extrapulmonary proximal airway (top and bottom panels). Boxed areas on top panels represent magnified areas presented in the middle and bottom panels. Scale bar: 200 μm (top panels), 50 μm (middle and bottom panels). (**B**) IHC for TRP63, KRT5, and SOX2 in control and BRD4 mutant E18.5 extra- and intrapulmonary proximal airways leading into the lungs. Boxes represent magnified areas in the right panels. Scale bar: 200 μm (left panels), 25 μm (right panels). (**C**) IHC for TRP63, KRT5, and SOX2 in control and BRD4 mutant E18.5 intrapulmonary proximal airways. Scale bar: 100 μm. (**D**) Quantification of the percentages of SOX2^+^ airway epithelium that are basal cells in trachea, extrapulmonary, and intrapulmonary proximal airways. Quantification data are represented as mean ± SEM. Two tailed *t* tests: **P ≤ 0.01, *n* ≥ 4.

Basal cells of the lung are multipotent stem cells that can give rise to cells of the proximal airways including secretory and ciliated cells (42–45). While in humans, basal cells extend from the trachea down into the lung respiratory bronchioles, they are restricted to the trachea and extrapulmonary proximal airways in mice (46). Given BRD4’s prominent role in proximal airway development, we asked whether loss of BRD4 led to defects in basal cell specification. We performed IHC on tissue sections with SOX2 and the mature basal cell marker, KRT5, and the immature and mature basal cell marker, TRP63. We analyzed 3 areas of the proximal airway including the trachea and extrapulmonary proximal airways (Figure 6A), the extra- and intrapulmonary proximal airway as it enters the lung (Figure 6, B and C). Surprisingly, we observed a significant increase in TRP63^+^KRT5^+^ basal cells in BRD4 mutant extrapulmonary proximal airways (Figure 6A), but they lost expression of KRT5 as they initiated entry into the hilum of the lung (Figure 6B) and became intrapulmonary proximal airways (Figure 6C), suggesting an immature basal cell phenotype. Additionally, we noted a rare cluster of TRP63^+^ only basal cells in the alveolar region in only a couple of the embryos from multiple litters (Supplemental Figure 6A). We quantified TRP63^+^ cells in all 3 areas of the proximal airway that revealed a significant increase in TRP63^+^ basal cells in the extrapulmonary and intrapulmonary proximal airways (Figure 6D).

### BRD4 regulates temporal acquisition of lung endodermal cell fate.

**Figure 7:**
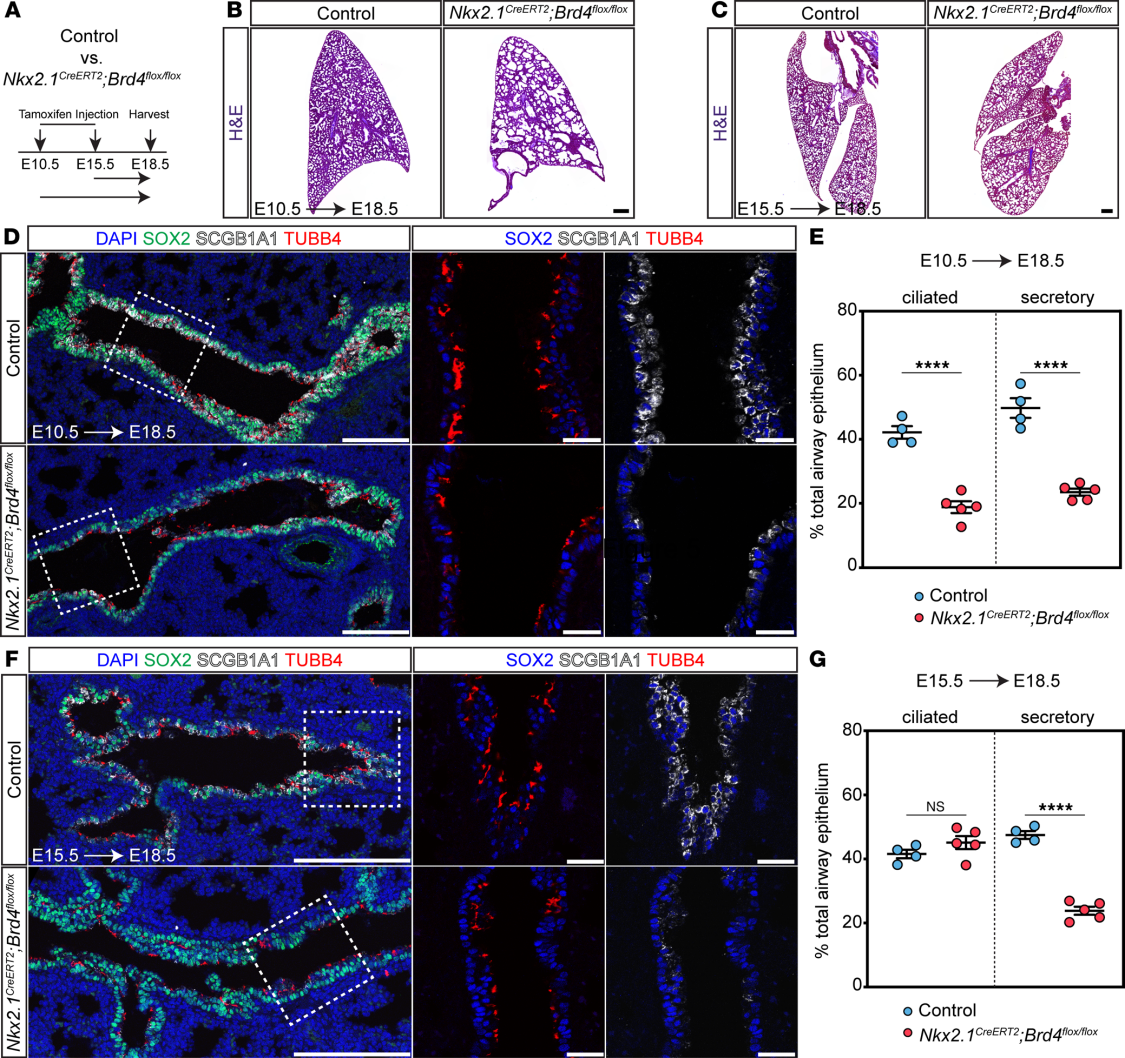
BRD4 regulates temporal acquisition of lung endodermal cell fate. (**A**) Experimental schematic indicating the time points of tamoxifen injection and harvest. (**B**) H&E staining of control and mutant lung at E18.5 with E10.5 tamoxifen induction. Scale bar: 200 μm. (**C**) H&E staining of control and mutant lung at E18.5 with E15.5 tamoxifen induction. Scale bar: 200 μm. (**D**) Left: IHC for SOX2, TUBB4, and SCGB1A1 in control and mutant E18.5 lungs with E10.5 tamoxifen induction. Scale bar: 100 μm. Dashed white boxes mark magnified regions shown in the middle and right panels. Middle: IHC for SOX2 and TUBB4 in magnified airway regions. Scale bar: 20 μm. Right: IHC for SOX2 and SCGB1A1 in magnified airway regions. Scale bar: 20 μm. (**E**) Quantification of the percentages of ciliated (left) and secretory (right) cells in the airway regions of control (blue) and mutant (red) lungs at E18.5 with E10.5 tamoxifen induction. Quantification data are represented as mean ± SEM. Two tailed *t* tests: ****P ≤ 0.0001, *n* ≥ 4. (**F**) Left: IHC for SOX2, TUBB4, and SCGB1A1 in control and mutant E18.5 lungs with E15.5 tamoxifen induction. Scale bar: 100 μm. Dashed white box marks magnified region shown in the middle and right panels. Middle: IHC for SOX2 and TUBB4 in magnified airway regions. Scale bar: 20 μm. Right: IHC for SOX2 and SCGB1A1 in magnified airway regions. Scale bar: 20 μm. (**G**) Quantification of the percentages of ciliated (left) and secretory (right) cells in the airway regions of control (blue) and mutant (red) lungs at E18.5 with E15.5 tamoxifen induction. Quantification data are represented as mean ± SEM. Two tailed *t* tests: ****P ≤ 0.0001, *n* ≥ 4.

Defects in branching morphogenesis can impair alveolar epithelial cell differentiation (47). Moreover, disruption of early proximal-distal patterning may compound later differentiation defects as early progenitor cells are lost. Thus, to determine whether cell specification defects in BRD4 mutants were affected by deficits in branching morphogenesis, we implemented a temporal deletion strategy in the lung endoderm. Using a pan-lung epithelial promoter–driven, inducible Cre recombinase mouse line (*Nkx2.1^CreERT2^*), we deleted BRD4 at E10.5 and E15.5, time points before and at the end of branching morphogenesis, respectively (Figure 7A). IHC for BRD4 protein confirmed a loss of expression in the epithelium in *Nkx2.1^CreERT2^ Brd4^fl/fl^* mutants (Supplemental Figure 7A). Assessment of morphological changes revealed that deletion of BRD4 at only E10.5 resulted in distal cystic structures and enlarged normal distal airspaces seen previously in *Shh^Cre^* *Brd4^fl/fl^* mutant embryos (Figure 7, B and C, and Supplemental Figure 7B). We employed IHC for both airway and alveolar epithelial markers to determine the effects of loss of BRD4 on cell specification at E10.5 and E15.5 (Figure 7, D–G, and Supplemental Figure 7, D–F). While alveolar epithelial specification was mildly altered after E10.5 deletion, we observed no changes in the numbers of AT1 or AT2 cells in mutants that were exposed to tamoxifen at E15.5 (Supplemental Figure 7, D–F). Additionally, only AT1 maturation defects were present after deletion of BRD4 at E10.5 (Supplemental Figure 8, A and B). Examination of defects in airway endoderm cell fate demonstrated significant decreases in the numbers of both SCGB1A1^+^ secretory and TUBB4^+^ multiciliated cells when deleted at E10.5 (Figure 7, D and E), but there was a decrease in only secretory cells after loss of BRD4 at E15.5 (Figure 7, F and G). These data indicate that loss of BRD4 in *Shh^Cre^* *Brd4^fl/fl^* mutants predominantly affects very early progenitor specification into both alveolar and airway lineages.

### BRD4 bromodomains inhibition disrupts hESC-derived lung endodermal airway cell fate.

**Figure 8:**
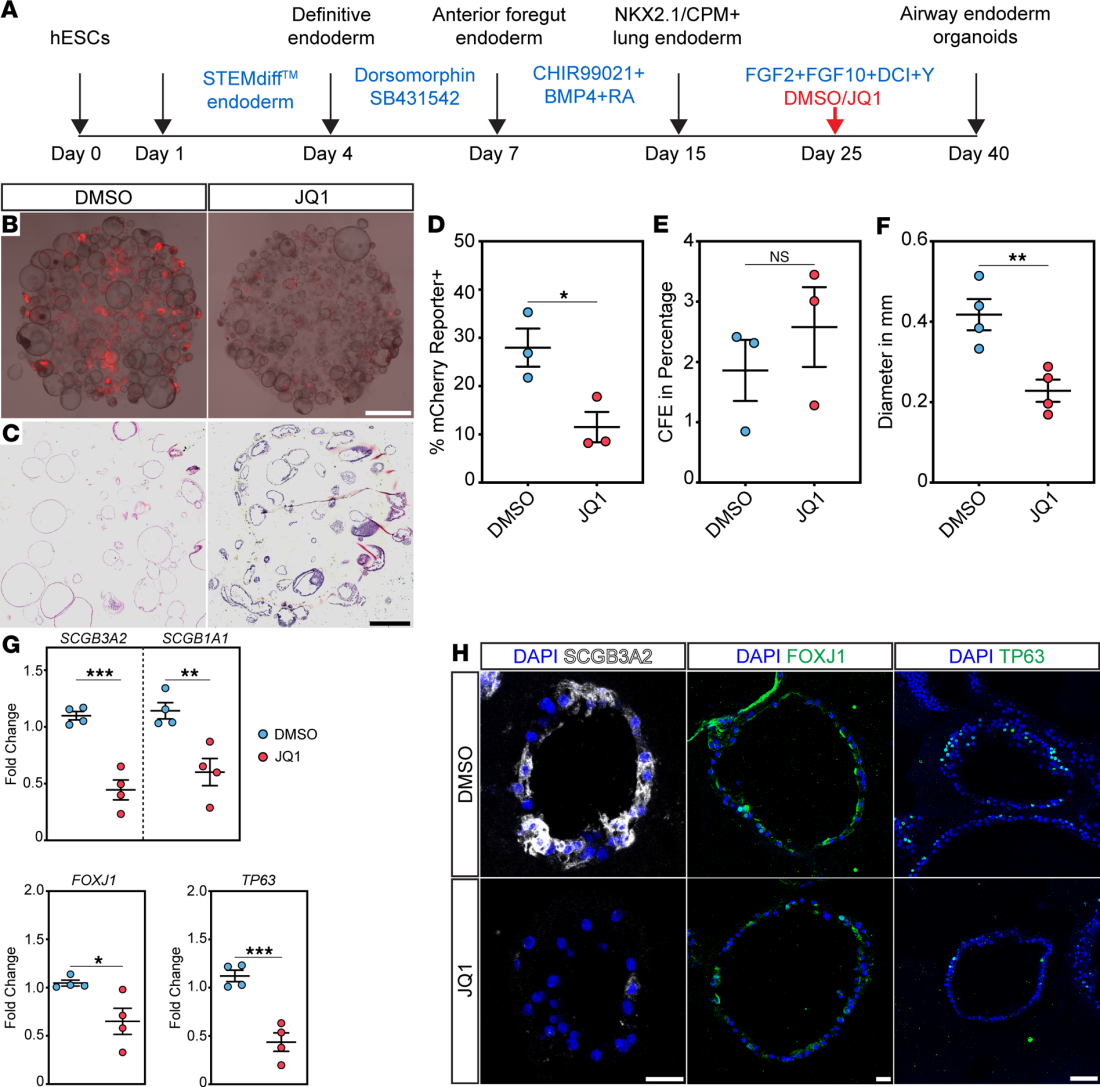
BRD4 bromodomains inhibition disrupts hESC-derived lung endodermal cell fate. (**A**) Experimental schematic indicating each step of deriving hESCs into airway organoids with components of the media. Red arrow indicates the day when JQ1 treatment begins. (**B**) Overlapped bright-field and fluorescent images of hESC-derived airway organoids with *SCGB3A2*^mCherry^ reporter on the day of harvest between DMSO (left) and JQ1 (right) treatment. Scale bar: 1 mm. (**C**) H&E of tissue sections of organoids treated with DMSO and JQ1. (**D**–**F**) Quantification of percentage of mCherry positive organoids (**D**), colony forming efficiency (**E**), and organoid diameter (**F**) between DMSO and JQ1 treatment. Each dot represents an individual differentiation experiment. Quantification data are represented as mean ± SEM. Two tailed *t* tests: *P ≤ 0.05, **P ≤ 0.01, *n* ≥ 3. (**G**) qPCR analysis of secretory cell marker genes, *SCGB3A2* and *SCGB1A1* (top), ciliated cell marker gene, *FOXJ1* (bottom left) and basal cell marker gene, *TP63* (bottom right) between DMSO (blue) and JQ1 (red) treatment. Each dot represents an individual differentiation experiment. Quantification data are represented as mean ± SEM. Two-tailed *t* test: *P ≤ 0.05, **P ≤ 0.01, ***P ≤ 0.001, *n* = 4. (**H**) IHC for (left) SCGB3A2, (middle) FOXJ1, and (right) TP63 in DMSO- and JQ1-treated hESC-derived airway organoids. Scale bars: 20 μm.

Given our findings demonstrating a prominent effect on secretory and ciliated cell specification, we asked whether there is a conserved role for BRD4 in mammalian airway endodermal specification. Additionally, emerging evidence indicates evolutionarily conserved mechanisms in lung development in mice and humans (48–53). Moreover, BET inhibitors are currently in trial for cancer and being considered in fibrosis, including lung fibrosis (31, 54), but their effects on normal or developing lung tissue are unknown. To determine the effects of BRD4 inhibition on human cells, we turned to an established hESC lung endodermal organoid differentiation model (48, 50). We used a hESC triple reporter line (RUES2-*SCGB3A2^mCherry^SFTPC^eBFP^AGER^eGFP^*) that fluorescently labels differentiated distal airway secretory cells, AT2 cells, and AT1 cells, respectively. To inhibit BRD4’s bromodomains reader function, we used the BET bromodomain inhibitor, JQ1, that competitively binds the BRD4 bromodomains with much higher affinity than the other BET family members such as BRD2 and BRD3 (55, 56). Following differentiation of hESCs into NKX2.1^+^ endoderm (57), cells were placed into Matrigel drops to form organoids and treated with airway endodermal differentiation media for a total of an additional 25 days (Figure 8A) (48, 50). Treatment with JQ1 at day 0 of differentiation resulted in complete inhibition of growth, a known role of BRD4 JQ1 (Supplemental Figure 9A). Therefore, JQ1 was added on day 10 of the 25-day airway differentiation protocol, several days after the first fluorescent reporter was detected (Supplemental Figure 9B). After imaging the organoids (Figure 8, B and C), quantification of organoid colony forming efficiency (CFE) and percent of organoids expressing fluorescently labeled cells demonstrated a significant decrease in *SCGB3A2^mCherry^*-positive organoids with JQ1 treatment compared with controls (Figure 8, D and E). Of note, we did not observe any *SFTPC^eBFP^*-positive or *AGER^eGFP^*-positive organoids, suggesting BRD4 has no role in the repression of alternative cell fates. JQ1 treatment was not associated with fewer colonies (Figure 8E), but there was a significant decrease in organoid size or diameter with treatment with JQ1 (Figure 8F), corroborating its role in proliferation. To confirm deficits in airway organoid differentiation, we performed qPCR for markers of proximal airway cells. We noted a marked decrease in expression of *SCGB3A2*, *SCGB1A1*, *FOXJ1*, and *TP63*, indicating differentiation defects in distal secretory and basal cells (Figure 8G). We corroborated these findings using IHC and observed similar expression defects in SCGB3A2, FOXJ1, TP63, and KRT5 (Figure 8H and Supplemental Figure 9C).

**Figure 9:**
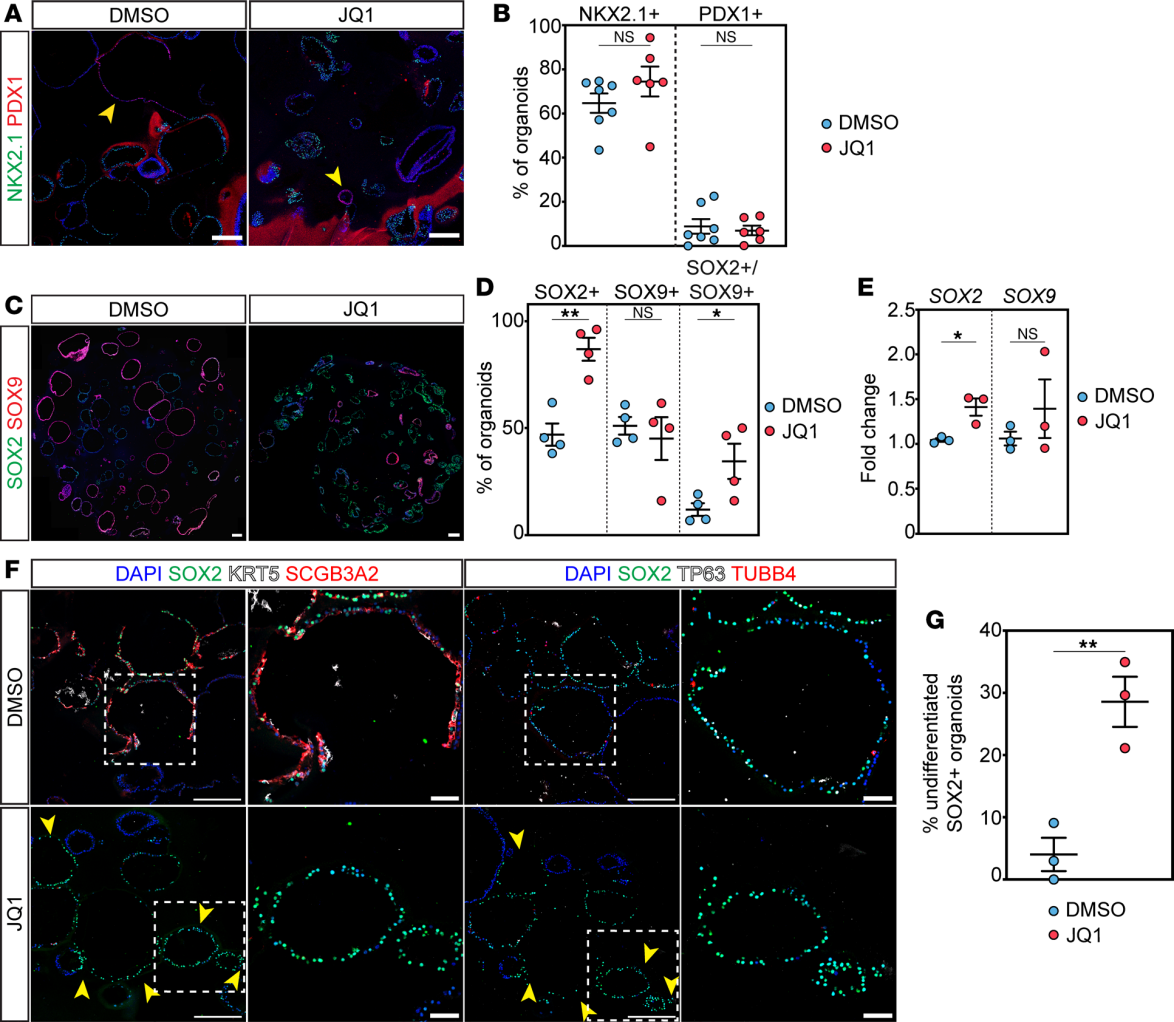
BRD4 bromodomain inhibition sequesters hESC-derived lung endodermal organoids in a SOX2 progenitor state. (**A**) IHC for NKX2.1 and PDX1 in DMSO- and JQ1-treated organoids. Scale bars: 100 μm). (**B**) Quantification of the percentages of NKX2.1^+^ or PDX1^+^ organoids between DMSO and JQ1 treatment. Quantification data are represented as mean ± SEM. Two-tailed *t* tests. *n* ≥ 6. (**C**) IHC for SOX2 and SOX9 in DMSO- and JQ1-treated organoids. Scale bar: 100 μm. (**D**) Quantification of the percentages of organoids containing cells that are SOX2^+^, SOX9^+^, and SOX2^+^SOX9^+^. Quantification data are represented as mean ± SEM. Two-tailed *t* test: *P ≤ 0.05, **P ≤ 0.01. *n* = 4. (**E**) qPCR analysis of organoids expressing *SOX2* or *SOX9*. Quantification data are represented as mean ± SEM. Two-tailed *t* test: *P ≤ 0.05. *n* = 3. (**F**) IHC for SOX2, KRT5, and SCGB3A2 (left 4 panels) and SOX2, TP63, and TUBB4 (right 4 panels) in DMSO- and JQ1-treated organoids. Boxed areas represent magnified areas shown on the panels to the right of each image. Scale bar: 200 μm, 50 μm (magnified boxes). (**G**) Quantification of the percentages of undifferentiated organoids. Quantification data represented as mean ± SEM. Two-tailed *t* test: **P ≤ 0.01. *n* = 3.

We first determined whether our differentiation defects were a result of a loss of lung cell identity and performed IHC for NKX2.1 and PDX1, a gene expressed in the developing posterior stomach, anterior duodenum, pancreatic buds, and the proximal extrahepatic biliary system (58). Quantification of the number of lung-specific and primitive gut organoids revealed that organoids were homogenous in expressing an organ-specific marker, and over 70% of all organoids expressed only NKX2.1 (Figure 9, A and B). While nearly 10% of organoids expressed PDX1, there were no differences between DMSO and JQ1 treatment (Figure 9B).

Given that most organoids were NKX2.1^+^, we determined whether these cells remained in an undifferentiated proximal airway state expressing SOX2 (Figure 9, C–E). Interestingly, control organoids contained nearly 50% of the organoids containing only SOX2^+^ cells or SOX9^+^ cells with very few containing both SOX2^+^ and SOX9^+^ cells (SOX2^+^SOX9^+^ organoids) (Figure 9, C and D). Oppositely, treatment with JQ1 significantly increased the number of SOX2^+^ cell containing organoids in addition to SOX2^+^SOX9^+^ organoids with no change in SOX9^+^ organoids. We confirmed these increases using qPCR for both *SOX2* and *SOX9*, demonstrating a significant increase in *SOX2* expression with no change in *SOX9* upon JQ1 treatment (Figure 9E). Additionally, we associated this increase in SOX2^+^ organoids with differentiated airway cell markers using IHC on sequential sections to stain the same organoid for multiple markers in the presence of SOX2 expression. While DMSO-treated organoids simultaneously expressed SOX2 and differentiated cell markers such as SCGB3A2, TUBB4, and TP63 or KRT5 (Figure 9, F and G), JQ1 treatment resulted in nearly zero organoids, with SOX2^+^ coexpressed with a differentiated airway cell marker, indicating that BRD4 is critical for differentiation and cell identity of airway epithelial cells.

To rule out cell differentiation defects related to increased cell death or a loss of proliferation, we performed IHC for cleaved caspase 3 and observed very little to no cell death in the organoids (Supplemental Figure 9D). We also assessed differences in proliferation via MKI67 expression. Surprisingly, we saw an increase in MKI67^+^SOX2^+^ cells within organoids with JQ1 treatment (Supplemental Figure 9, E and F). Given that MKI67 is expressed in a gradient across G1, S, and G2M and that JQ1 treatment arrests cells in G1, we also examined proliferation with staining for proliferating cell nuclear antigen (PCNA) and minichromosome maintenance complex component 5 (MCM5) that mark cells predominantly in S phase and phospho-histone H3 (PHH3), a marker for mitosis (Supplemental Figure 9, G–L) (59, 60). Consistent with MKI67 expression, we observed an increase in both PCNA and MCM5 expression in SOX2^+^ cells in organoids with JQ1 treatment (Supplemental Figure 9, G–J). With IHC for PHH3, we noted very few cells positive for PHH3 (Supplemental Figure 9K). We quantified the number of organoids that had a PHH3^+^ cell and noted no differences between DMSO and JQ1 treatments (Supplemental Figure 9L).

## Discussion

In this study, we determined that BRD4 is a critical regulator of lung development. In mice, loss of BRD4 results in perinatal lethality accompanied by abnormal branching morphogenesis, proximal-distal patterning, disruption of epithelial-mesenchymal crosstalk, and cell differentiation and maturation within the lung. Moreover, inhibition of BRD4 bromodomains revealed similar defects in human ESC endodermal differentiation into airway lineages. Together, these findings report a critical and multifunctional role for BRD4 in lung development across species and suggest that further investigation is warranted to evaluate the role of BRD4 in adult disease and clinical treatment contexts.

Multiple loss-of-function genetic studies have identified cystic distal lung structures as a major phenotype (5–7, 11, 35, 61, 62). However, no clear, comprehensive mechanism driving this phenotype exists. Prior studies demonstrate that a SHH/BMP4/FGF signaling loop plays an important role in branching morphogenesis (34–37), but interrogation of this pathway in most studies with cystic distal lung structures revealed no changes in the endodermal-mesodermal patterning by SHH, BMP4, and FGF10. In our studies, BRD4 deficiency in the endoderm results in a reduction of distal tip SHH expression concomitant with an increase and expansion of BMP4 and FGF10 as measured by multiplex RNA FISH, qPCR of FGF10 and SHH target genes in the mesenchyme, and RNA-seq of the epithelium at E12.5. This was associated with branching defects and proximal-distal patterning. These findings suggest a BRD4/SHH/BMP4/FGF10 interaction not only mediates epithelial-mesenchymal communication but also ensures appropriate expansion of proximal, SOX2^+^ endoderm during morphogenesis. Whether this interaction is direct is unclear, but BRD4 is a multifunctional tool regulating both transcriptomic and epigenetic mechanisms. Therefore, future studies are warranted to not only interrogate how BRD4 regulates gene expression but also to explore the role of additional pathways and transcriptional programs identified in our study, including but not limited to retinoic acid, NOTCH signaling, HOX genes, and forkhead domain transcription factors.

Beyond regulation of morphogenesis, BRD4 is an important regulator of cell specification and differentiation during lung development. Besides its implication in heart disease and cancer, BRD4 plays a broad role in embryogenesis (54, 63). BRD4 orchestrates implantation and embryonic growth, maintains stem cell identity, regulates fat and muscle development, and promotes neural crest and other progenitor cell differentiation (25, 27, 28, 33). We observed a cell autonomous defect in specification, differentiation, and maturation of both proximal airway and distal alveolar epithelial cell subtypes, indicating a global role for BRD4 in lung cell specification in vivo. Interestingly, loss of secretory cells in the conducting airway is more pronounced, a paradigm also observed in our hESC lung endodermal organoid differentiation model. Similar deficits in secretory cells after loss of BRD4 have been observed in the small intestine, suggesting a conserved mechanism for secretory cells specification (64).

However, there was a discrepancy in mouse and human airway basal cell differentiation from early lung progenitors. We observed an increase in basal cells in mice and a decrease in basal cells in human ESC differentiation that could arise from several potential mechanistic differences. Our human ESC differentiation used JQ1 treatment 10 days into differentiation to avoid the significant growth inhibition we saw, with inhibition at day 0 and an attempt to time BRD4 inhibition with the earliest SCGB3A2^mCherry^ reporter detection after day 8. Thus, we may be affecting loss of cell identity rather than differentiation in the human ESC system, but we believe it could be both, as we detected only a few cells with reporter expression at days 8–10,with airway differentiation being a progressive process. Moreover, we see a significant increase in SOX2^+^ organoids, suggesting failure of differentiation of SOX2^+^ lung progenitor cells, and we did not see a difference in loss of lung identity as measured by NKX2.1 and PDX1 expression, a marker of developing posterior stomach, anterior duodenum, pancreatic buds, and the proximal extrahepatic biliary system, with JQ1 treatment (58). Additional possibilities include that the hierarchy of lung stem cell development may differ between mice and humans. Basal cells in mice and humans differ in their spatial localization in that basal cells are almost entirely found in the trachea and proximal bronchi in mice but can extend down into the small airways in humans. Therefore, it is conceivable that differentiation programs for airway cells in various mammalian models may vary. For example, in human ESCs, we may be inhibiting only differentiation of SOX2^+^ stem cells directly into airway lineages, whereas in mice, we are inhibiting not only differentiation of SOX2^+^ lung stem cells but also subsequent or simultaneous differentiation of TRP63^+^ basal cells.

JQ1 treatment most frequently results in inhibition of proliferation, but this is predominantly in the setting of tumorigenesis and transformed cell lines. While rare, there are several studies indicating that JQ1 can promote proliferation in hematopoietic stem cells in vitro and hepatoblast-like cells during liver regeneration in vivo (65, 66). In our model, we observed an increase in proliferation that may reflect the cell state induced by JQ1 treatment as cells remained or dedifferentiated into SOX^+^ early multipotent lung stem cells. Alternatively, JQ1 promoting expression of multiple markers of cell proliferation in our in vitro model may result from its known function to arrest cells in G1 (67). While MKI67, PCNA, and MCM5 mark S phase, they can also be expressed during late G1 phase (59, 60).

Whether BRD4 is a direct regulator of the genes important for specification of every type of lung epithelial cell is unclear. It is possible that early defects in proximal-distal patterning and branching disrupt alveolar differentiation in development (47). Using an inducible Cre recombinase, we noted deficits in airway epithelial cell differentiation with deletion as late as E15.5 during development. This occurred toward the end of branching morphogenesis and indicates a direct role for BRD4 in airway epithelial cell differentiation throughout development. BRD4’s role in alveolar epithelial differentiation is less clear, as late deletion had no effect on differentiation. These findings suggest that BRD4 functions predominantly in early stem cell fate and less so in later progenitor cell specification.

These studies provide evidence of the role of the epigenetic factor, BRD4, in lung development. Like previous studies on the epigenetics of lung development, there are global defects including deficits in branching morphogenesis, proximal-distal patterning, and progenitor cell specification and cell maturation (4, 7, 9, 11). Current studies indicate that BRD4 is a prominent participant in the progression of cancer, heart disease, and lung fibrosis (54, 68). Our data demonstrating BRD4 as a master regulator of lung development are important, given ongoing clinical trials for cancer and fibrosis. Fetal programs are often reactivated for repair processes. In the setting of BRD4 inhibition, loss of its function could be detrimental to repair after injury. Thus, future studies exposing its role in adult injury and repair are critical.

## Methods

### Sex as a biological variable.

Embryos were genotyped for Sry to ensure equal male and female samples for each experiment. There were no detectable differences noted between sexes.

### Mouse lines.

Information related to the generation and genotyping of the following mouse lines has been previously described: *Shh^Cre^* (Jackson Laboratory, stock # 005622) *Nkx2.1^CreERT2^* (Jackson Laboratory, stock # 014552), *Brd4^fl/fl^*, and *R26R^EYFP^* (Jackson Laboratory, stock # 007903). The *Brd4^fl/fl^* mice were provided by Keiko Ozato’s laboratory (National Institute of Child Health and Child Development, Bethesda, Maryland) (33). Matings were set up to generate *Shh^Cre/+^* *Brd4^fl/fl^* *R26R^EYFP/+^* and *Nkx2.1^CreERT2/+^* *Brd4^fl/fl^* *R26R^EYFP/EYFP^* lines and maintained on a mixed C57BL/6 and CD1 background. Experiments were performed with a minimum of 3 animals per condition of mixed sex, and littermate controls were included. Controls included Cre negative and *Shh^Cre^* and *Nkx2.1^CreERT2^* WT and heterozygous BRD4 mutant embryos.

### Tamoxifen administration.

Tamoxifen (Sigma-Aldrich) was dissolved in a concoction of 100% ethanol and corn oil with 10% final volume of ethanol and 90% final volume of corn oil. To temporally control the deletion of *Brd4* during mouse development, pregnant dams were orally gavaged with tamoxifen at E10.5 and E15.5 at a concentration of 200 mg/kg of mouse.

### Tissue processing and histology.

Embryonic lungs were harvested in cold PBS, placed into 2% paraformaldehyde, and fixed for either 5–6 hours (wholemount) or overnight (IHC) at 4°C. After fixation, lungs were washed with PBS at least 4 times, dehydrated in a series of ethanol washes (30%, 50%, 70%, 95%, and 100%), embedded in paraffin wax, and sectioned at a thickness of 6 μm. H&E staining was performed on slides with maximum surface area to observe for gross morphological changes.

### IHC and RNA FISH.

IHC staining was performed as previously described (18, 69, 70). The slides were then blocked using 5% donkey serum and incubated with primary antibody at 4°C overnight in predetermined concentrations (Supplemental Table 4). The presence of relevant proteins was visualized using Alexa Fluor secondary antibodies (Supplemental Table 5). After secondary antibody staining, slides were mounted in Vectashield Antifade Mounting Medium (H-1000, Vector Laboratories) and imaged using Leica DMi8 confocal microscope.

RNA FISH was performed using the RNAscope Multiplex Fluorescent V2 Assay (323100, Advanced Cell Diagnostics [ACDBio]) according to the manufacturer’s instructions. RNA 3-plex negative control probe (DapB) and mouse specific 3-plex control probe (Polr2a) were used for control sections. The RNA target probes included *Shh*, *Bmp4*, *Fgf10*, *Etv5*, and *Fgfr2*, and mRNA transcripts were visualized using OPAL fluorophore reagents (Opal 540 FP1494001, Opal 570 FP11488001, or Opal 650 FP1496001, Akoya Biosciences) and mounted with Prolong Gold Antifade Mountant (P36930, Thermo Fisher Scientific).

### Wholemount immunofluorescence.

Wholemount samples were prepared and stained as previously described (69). E12.5 or E15.5 lungs were stained with SOX2 (goat, R&D, AF2018, 1:50) and SOX9 (rabbit, Abcam, Ab185966, 1:200) in 5% donkey serum and 0.5% Triton X-100 in PBS (0.5% PBST) for 2–4 hours at room temperature and then overnight at 4°C with gentle rotation. Following sample washes, the lungs were stained with secondary antibodies (donkey anti-goat, and donkey anti-rabbit, 1:250) in 5% donkey serum and 0.5% PBST.

### Ex vivo lung explant assay.

Lungs were harvested and cultured for 72 hours as previously described (7, 71). Briefly, E12.5 lungs were dissected into cold PBS, washed at least twice in DPBS supplemented with Antibiotic-Antimycotic and once in DMEM/F12 supplemented with Antibiotic-Antimycotic, and placed onto 1 μm–pore transwell inserts in DMEM/F12 supplemented with Antibiotic-Antimycotic. PMA (3μM, Tocris Bioscience, 4551) and DMSO treatment were mixed with culture media, added to predetermined lung explants, and refreshed every 24 hours. Lungs were imaged using a Leica DMi8 Thunder widefield microscope at 0 hour, 24 hours, 48 hours, and 72 hours.

### qPCR analysis.

RNA was isolated using Trizol (Invitrogen) reagent and purified using the RNA PureLink micro kit (Invitrogen). cDNA was synthesized from RNA using the SuperScript IV First-Strand Synthesis System (Thermo Fisher Scientific, 18091050) according to the manufacturer’s instructions. qPCR was performed with previously published primer sets (Supplemental Table 6) and PowerUp SYBR Green Master Mix (Applied Biosystems, A25742) (48).

### Distal airway/saccule area and diameter measurements.

Morphometric analysis of distal airway/saccule area and diameter was performed on H&E images of distal areas acquired using a Leica Thunder widefield microscope. Areas and diameters were extracted with previously described MATLAB software (72). For each mouse, the mean area and diameter were calculated from at least 6 randomly selected 20× fields.

### Quantification of branching points.

The branching defect analysis was performed on wholemount images of E12.5 lung buds obtained using a Leica thunder widefield microscope. Based on the Sox9 staining of the branches stemming from the 2 primary bronchi, we counted the number of branching points in FIJI.

### Quantification of ex vivo lung explant.

The area of SOX2 expression, tip area, and tip number were quantified based on wholemount IHC staining of SOX2 and SOX9. SOX9^+^ distal lung tips were manually counted across the entire *z* stack using the cell counter plugin in FIJI. Due to the oval shape of the tips, straight lines were drawn across the shorter minor axis of the tips to measure the tip thickness in FIJI. The thickness of over 50 lung tips was measured and averaged for each lung explant. For area measurement of SOX2 expression, images of SOX2 staining were turned into binary using the threshold automatically calculated within FIJI. Afterward, areas of SOX2 expression were selected using the wand tool, added onto the ROI manager, and measured.

### Quantification of in vivo epithelial cell proliferation.

To quantify proliferative epithelial cells, we adopted our previously established procedures using EdU staining (69). Briefly, pregnant dams were injected i.p. with 50 gm/kg of EdU, and the embryos were harvested 4 hours following the injection. After performing the click chemistry-based EdU proliferation assay (Click-iT Plus EdU Alexa Fluor 647, C10640, Thermo Fisher Scientific). The percentages of proliferative cells in proximal and distal airways were counted using the cell counter plugin in FIJI based on SOX2^+^EdU^+^ and SOX9^+^EdU^+^ respectively.

### Quantification of capillary EC number and density.

Capillary defects were characterized as described previously (69, 70). IHC staining for pan-endothelial marker ERG (mouse, ab214341, Abcam, 1:100), capillary and venous endothelial marker EMCN (rat, 14-5851-82, Invitrogen, 1:100), and CAR4 (goat, AF2414, R&D Systems, 1:100). At least 5 distal lung images per embryo were randomly captured and then processed using Imaris software (version 10.0.0). The relative loss of endothelial cells was calculated based on the ratio of ERG^+^ cells in relation to DAPI^+^ cells in the field. Quantification of capillary density was performed based on previously determined procedures (69). Volume of the whole tissue (DAPI), capillary EC (EMCN), and CAR4^hi^ capillary EC (CAR4) was measured, and the capillary density and CAR4^hi^ capillary density were calculated by dividing the capillary EC volume and CAR4^hi^ capillary EC volume by the total lung volume respectively.

### Cell counting.

Cell counting analysis was performed based on a previously established protocol (73). The cells of interest were manually counted based on signal colocalization using the cell counter plugin in FIJI. For a given stain, at least 500 cells or 5 confocal stacks captured at 40× magnification selected images were counted per animal. For quantification of AT1, AT2, and HOPX^+^SFTPC^+^ cell percentages, cell counts were normalized to the number of NKX2.1^+^ cells. For quantification of secretory and ciliated cell percentages, cell counts were normalized to the number of SOX2^+^ cells. For quantification of basal cell percentages, cell counts were normalized to the number of SOX2^+^ cells.

### hESC lung organoids differentiation, histology, and RNA isolation.

The human ES cell triple reporter, REUS2 SCGB3A2^mCherry^SFTPC^eBFP^AGER^eGFP^ cell line was developed and provided by the laboratory of Maria Basil. Human ESCs were differentiated into lung airway cell lineages using a previously described protocol (48–50). Briefly, NKX2.1^+^ lung progenitor cells were derived from ES cells by inducing definitive endoderm differentiation using the STEMdiff Definitive Endoderm Kit (StemCell Technologies) over a period of 72 hours. The definitive endoderm was then dissociated and transferred in small clumps to Matrigel-coated tissue culture plates, where they were cultured in complete serum-free differentiation medium (cSFDM). NKX2.1^+^ primordial lung progenitors were subsequently obtained. These lung progenitors were sorted based on counts per million(CPM) expression (Wako Chemicals) (74) and then replated in 3-dimensional, growth-factor-reduced Matrigel at a density of 50,000 cells per 100 μL. The cultures were maintained at 37°C for 20 minutes to allow the Matrigel to solidify, after which additional medium was added to cover the droplets.

For the development of airway organoids, lung progenitors were cultured for 25 days in airway medium containing cSFDM, FGF2 (250 ng/mL, R&D Systems), FGF10 (100 ng/mL, R&D Systems), dexamethasone (50 nM, Sigma-Aldrich), 8-bromoadenosine 3′,5′-cyclic monophosphate sodium salt (100 nM, Sigma-Aldrich), IBMX (100 mM, Sigma-Aldrich), and Thiazovivin (2 mM, Selleckchem). JQ1 (100 nM, MedChemExpress) was added to the airway media on day 10, with DMSO as the control. The media were replaced every 48 hours until harvested.

For IHC, Matrigel droplets containing organoids were embedded in Histogel (Thermo Fisher Scientific, HG4000012), fixed in 2% PFA for 30 minutes, then processed, paraffin embedded, sectioned, IHC stained as described above. For RNA isolation, the organoids were transferred to a 1.5 mL Eppendorf tube and washed with ice cold PBS at least 4 times to remove the Matrigel before extracting the RNA, making the cDNA, and running the qPCR analysis as described above.

### Quantification of mCherry^+^ organoids, CFE, and organoid diameter.

To visualize the fluorescent reporters, organoids were imaged directly within Matrigel droplets using the Leica DMi8 Thunder system on the day of harvest. Images were acquired at 4× magnification. The total number of organoids and number of mCherry^+^ organoids were manually counted on bright-field and fluorescent reporter using the cell counter plugin in FIJI. CFE was calculated as the ratio of the number of organoids formed to the total number of cells seeded per Matrigel droplet. To quantify the diameter of the organoid, the straight-line tool in FIJI was used to draw the diameter of the organoids and measure the distance. For each droplet, 40 randomly selected organoids’ diameters were averaged.

### Quantification of NKX2.1^+^ and PDX1^+^ organoids.

For all organoid quantifications, IHC tile scan images of entire organoid section across the Matrigel drop were captured using a Leica DMi8 confocal microscope at 40× magnification and processed in FIJI. An NKX2.1^+^ or PDX1^+^ organoid is defined as an organoid containing any cell with expression of NKX2.1 or PDX1, respectively. The NKX2.1^+^ and PDX1^+^ organoid counts were normalized to the total number of organoids by identified by DAPI staining.

### Quantification of SOX2^+^, SOX9^+^, and SOX2^+^SOX9^+^ organoids.

A SOX2^+^ or SOX9^+^ organoid is defined as an organoid containing any cell with expression of SOX2 or SOX9, respectively. A SOX2^+^SOX9^+^ organoid is defined as an organoid containing both SOX2^+^ cells and SOX9^+^ cells. The SOX2^+^, SOX9^+^, and SOX2^+^SOX9^+^ organoid counts were normalized to the total number of organoids identified by DAPI staining.

### Quantification of undifferentiated SOX2^+^ organoids.

To include all 4 (KRT5, SCGB3A2, TP63, and TUBB4) lung airway epithelial cell markers, serial sections of lung organoids were used. A SOX2^+^ organoid is defined as an organoid containing any cell with expression of SOX2. An undifferentiated SOX2^+^ organoid is defined as an organoid that contains SOX2^+^ cells without any differentiated airway epithelial cell markers previously mentioned. The undifferentiated SOX2^+^ organoid counts were normalized to the total number of SOX2^+^ organoids.

### Quantification of organoid proliferation.

For each proliferation marker (MKI67, PCNA, MCM5, and PHH3), a proliferating SOX2^+^ cell is defined by the colocalization signals of both SOX2 and the proliferation marker. The proliferating airway cell counts were normalized to the number of SOX2^+^ cells. In the case of PHH3, due to the small number of cells undergoing mitosis, we quantified the percentage of total organoids with a PHH3^+^SOX2^+^ cell. A SOX2^+^ organoid is defined as an organoid containing any cell with positive signals for SOX2. The PHH3^+^SOX2^+^ organoid counts were normalized to the total number of SOX2^+^ organoids.

### scRNA-seq analysis.

Lungs were dissected and dissociated for scRNA-seq as previously described (18, 69). Approximately 16,500 EPCAM^+^ cells from pooled mice population of *Shh^Cre^* *Brd4^fl/+^* heterozygous control or *Shh^Cre^* *Brd4^fl/fl^* homozygous KO mice were loaded into individual lanes of a 10X Genomics 3′ gene expression scRNA-seq assay (v3.1) chip (10X Genomics) to target recovery of 10,000 live cells.

Reads were aligned to the GRCm38/mm10 mouse genome, and unique molecular identifiers (UMIs) were counted using STAR-solo (75). Downstream scRNA-seq processing, including filtering, normalization, cell type identification, cluster identification, and differential gene expression analysis, was performed using scanpy (76). Gene expression counts were normalized to ln(1+CPM) using the sc.pp.lop1p function. Cells were filtered to only include those with greater than 1,000 unique genes detected and fewer than the ninety-fifth percentile (4714 genes). Likewise, cells with more than the ninety-fifth percentile of percentage of UMIs derived from mitochondrial mRNAs (11.5%) were excluded. Cell doublets were identified and removed using the scVI-tools implementation of the SOLO doublet detection algorithm (77, 78).

Cells were assigned to cell types based on the expression of canonical marker genes for lung AT1, AT2, secretory, and multiciliated cell types. Differential expression testing was performed using the sc.tl.rank_genes_groups function with method=“wilcoxon”. Differentially expressed genes include only those with a log_2_ fold change (LFC) of magnitude greater than 1 and were filtered to remove those genes associated with sex chromosome regulatory mechanisms, pseudogenes, mitochondrial genes, and ribosomal genes.

ORA of differentially expressed genes was performed using gProfiler. MSigDB hallmark gene set scoring was performed across subsets of cells by first *z* scoring the ln(1+CPM) gene expression values of those cells and then calling sc.tl.score_genes function from scanpy on genes contained in each gene set (79, 80).

### Statistics.

Unpaired, 2-tailed *t* tests or 1-way ANOVA were used to detect significant statistical differences in quantification analyses. All statistical analyses were performed using Prism 9.0 with the following statistical significance: *P ≤ 0.05, **P ≤ 0.01, ***P ≤ 0.005, ****P ≤ 0.001. Specific *n* values are listed in the figure legend. Each point on the graph represents a single biological replicate obtained through averaging cell counts from multiple regions of a single animal or multiple droplets of a single-cell culture experiment.

### Study approval.

Animal procedures were ethically performed in compliance with the *Guide for the Care and Use of Laboratory Animals* (National Academies Press, 2011). These studies were approved under the guidance of the University of Pennsylvania and Children’s Hospital of Philadelphia IACUC.

### Data availability.

Raw and processed next-generation sequencing data sets have been uploaded to the NCBI GEO database (https://www.ncbi.nlm.nih.gov/geo/). The GEO accession ID for scRNA-seq data is GSE295345 and GSE312831 for the E12.5 bulk RNA-seq data. All Supporting data values associated with the main manuscript and supplement material are included in a Supporting Data Values file.

## Author contributions

Conceptualization was carried out by DBF, DCL, MCB, RJ, and HW. Resources were from DBF, MCB, LRY, and RJ. Investigations were completed by HW, PC, DCL, SP, KKQ, MK, ANL, ATM, AJ, ML, AAF, S Suresh, S Shirazi, DBF, and SNM. Analyses were performed by HW, PC DCL, SP, S Suresh, S Shirazi, JMSS, SNM, and DBF. Visualization was done by HW, DCL, PC, SP, SNM, and DBF. Writing the original draft was done by HW, DCL, SNM, and DBF. Review and editing were done by DCL, HW, PC, LRY, MCB, RJ, and DBF.

## Funding support

This work is the result of NIH funding, in whole or in part, and is subject to the NIH Public Access Policy. Through acceptance of this federal funding, the NIH has been given a right to make the work publicly available in PubMed Central.

- NIH grants K08 HL140129 and R56 HL167937 (DBF)
- NIH grant R35 HL166663 (RJ)
- NIH grants K08 HL143051 and R01 HL168556 (JMSS)
- NIH grant K08 HL163398 (MCB)
- NIH grants K24 HL143281 and R01 HL119503 to LRY
- Parker B. Francis Foundation (DBF and JMSS)
- Burroughs Wellcome Foundation Career Award for Medical Scientists (RJ and MCB),

## Supplementary Material

Supplemental data

Supporting data values

## Version 1 02/03/2026

In-Press Preview

## Version 2 03/09/2026

Electronic publication
