# Supplementary material for: BRD4 promotes endodermal cell fate during mammalian lung development
Source: JCI Insight. 2026 Feb 3;11(5):e194683. doi: 10.1172/jci.insight.194683 (PMC13041681; doi:10.1172/jci.insight.194683)
Supplement: Supplemental data [file jciinsight-11-194683-s116.pdf]

Supplemental Figure 1

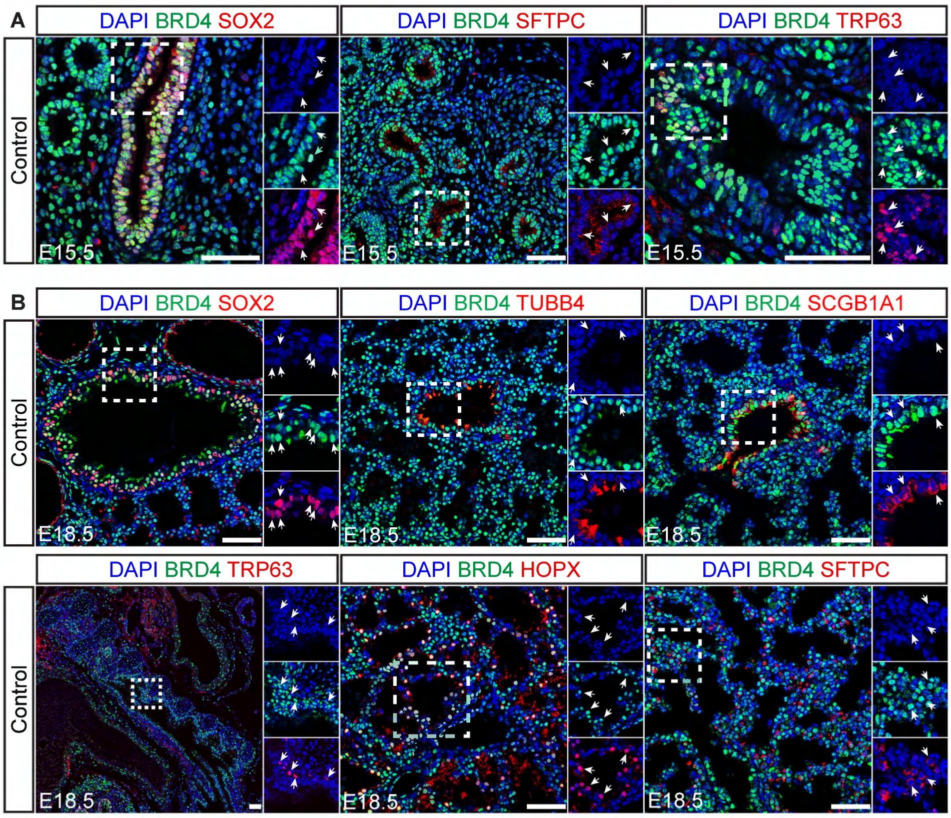

C

Perinatal survival			
Genotype	Total	Observed%	Expected%
Brd4$^{flox/+}$ or *Brd4*$^{flox/flox}$	35	66%	50%
Shh^{Cre};*Brd4*$^{flox/+}$	18	34%	25%
Shh^{Cre};*Brd4*$^{flox/flox}$	0	0%	25%

D

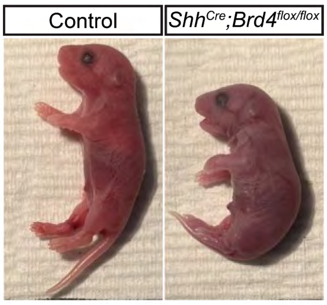

E

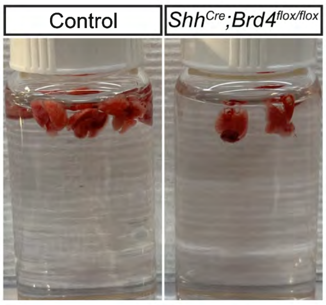

Supplementary Figure S1. Expression of BRD4 across development and perinatal lethality in BRD4 mutant mice. (A) Immunohistochemistry (IHC) for BRD4, SOX2, SFTPC, and TRP63 in control E15.5 embryonic lungs. Boxed areas represent magnified images presented to the right of each large panel (scale bar: 50 µm). White arrows point at cells expressing both BRD4 and the respective epithelial cell marker. (B) IHC for BRD4, SOX2, TUBB4, SCGB1A1, TRP63, HOPX, and SFTPC in control E15.5 embryonic lungs. Boxed areas represent magnified images presented to the right of each large panel (scale bar: 50 µm). White arrows point at cells expressing both BRD4 and the respective epithelial cell marker. (C) Table showing perinatal survival from 6 litters of a Shh-^{Cre};Brd4$^{flox/+}$ male crossed with Brd4$^{flox/flox}$ females. (D) Control and mutant pup images just after birth. (E) Lung float test for control and mutant mice.

Supplemental Figure 2

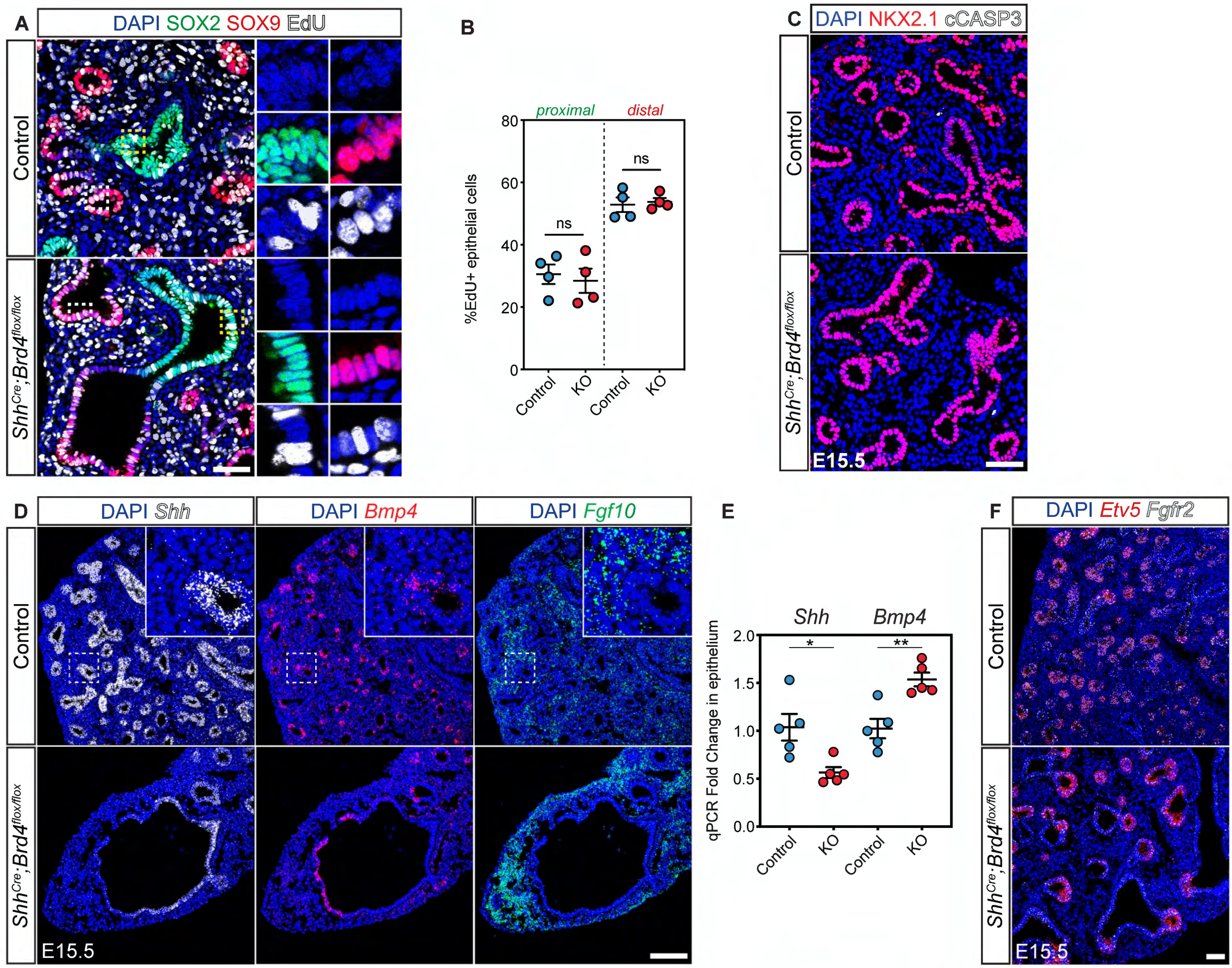

Supplementary Figure S2. Cellular changes associated with BRD4 mutant mice. (A) IHC for SOX2 and SOX9 and staining for EdU at E15.5. Dashed boxes mark zoomed in areas of proximal (yellow) and distal (white) endoderm (scale bar: 50 μm). (B) Quantification and comparison of proximal (SOX2+EdU+/SOX2+) and distal (SOX9+EdU+/SOX9+) endodermal EdU incorporation at E15.5 in control versus mutant lungs as shown in panel (A). Quantification data are represented as mean ± SEM. Two-tailed t tests: ns: not significant, n = 4. (C) IHC for NKX2.1 and cleaved caspase 3 (cCASP3) in control and mutant E15.5 lungs (scale bars: 50 μm). (D) RNA FISH for *Shh*, *Bmp4*, and *Fgf10* in control and mutant E15.5 lungs (scale bar: 100 μm). (E) qPCR analysis of *Shh* and *Bmp4* in control and mutant E15.5 lung epithelium. Quantification data are represented as mean ± SEM. Two-tailed t test: *p ≤ 0.05, **p ≤ 0.01, n = 5. (F) RNA FISH of *Etv5* and *Fgfr2* in control and mutant E15.5 lungs (scale bars: 50 μm).

Supplemental Figure 3

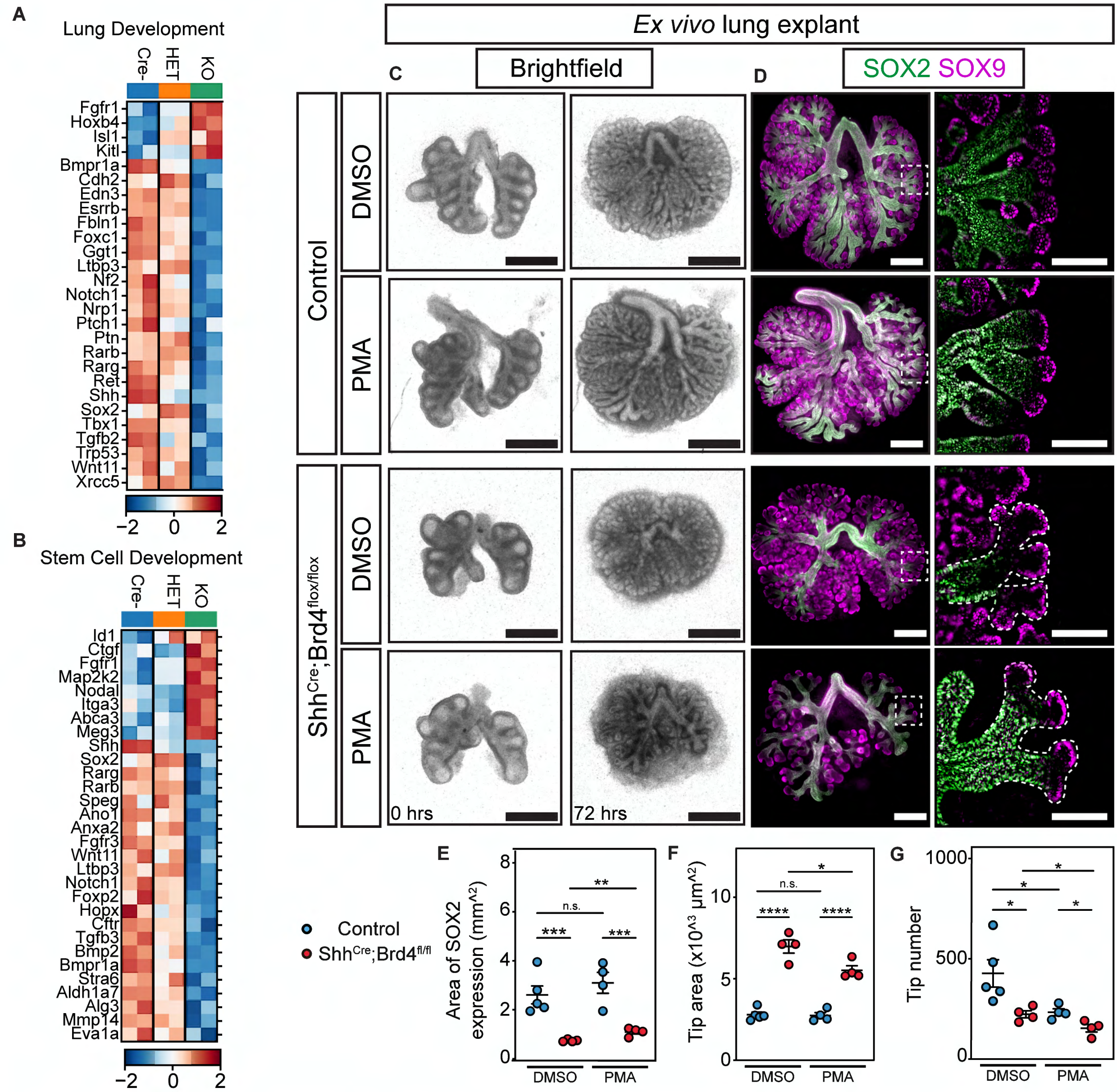

Supplementary Figure S3. SHH activation partially rescues BRD4 mutant cystic distal airway structure phenotype. (A) Matrixplots of differentially expressed lung development genes by RNA sequencing of E12.5 lung epithelium. (B) Matrixplots of differentially expressed stem cell development genes by RNA sequencing of E12.5 lung epithelium. (C) Ex vivo lung explant brightfield images of control and mutant lungs at (left) E12.5 and (right) E15.5 with treatments: DMSO and Purmorphamine (PMA) (scale bar: 500 µm). (D) Left: wholemount IHC for SOX2 and SOX9 after 3 days of ex vivo culture (scale bar: 500 µm). Dashed white boxes indicate branching tips in control and mutant lungs. Right: magnified images of branching tips, dashed white lines outline a distal airway (scale bar: 250 µm). (E-G) Quantification and comparison of (E) area of SOX2 expression, (F) tip area, and (G) tip number between control and mutant lungs treated with DMSO and PMA. Quantification data are represented as mean ± SEM. Two tailed t-tests: ns.: not significant, *$p \le 0.05$, **$p \le 0.01$, ***$p \le 0.001$, ****$p \le 0.0001$, $n \ge 4$.

Supplemental Figure 4

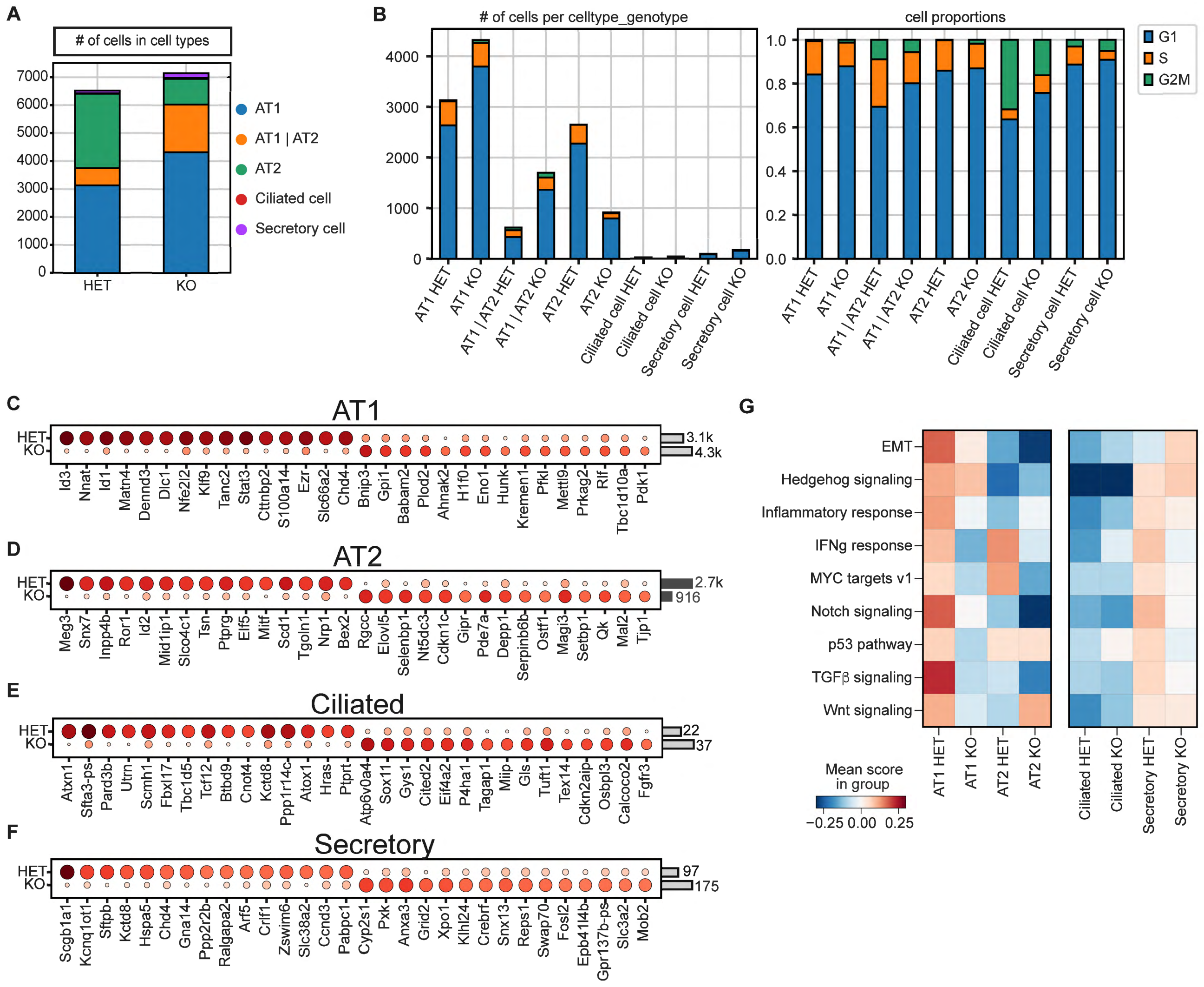

Supplementary Figure S4. Alterations in gene expression in BRD4 mutant epithelial cells. (A) Cell compositional changes in BRD4 heterozygous and homozygous knockout lungs based on cell number. (B) Bar graphs demonstrating cell cycle phase assessment for cells in combined genotype and cell type clusters by total number of cells (left) and cell proportion (right) in BRD4 heterozygous lungs and homozygous knockout lungs. (C-F) Dot plots of the top 15 upregulated and top 15 downregulated differentially expressed genes in heterozygous control and homozygous BRD4 mutant AT1s (C), AT2s (D), ciliated (E), and secretory (F) cells. Dot size indicates the proportion of cells within a cluster expressing a gene, and color intensity indicates the relative expression level. (G) Heatmap representing expression levels of signaling and response pathway hallmark scores from AT1 and AT2s (left panel) and ciliated and secretory cells (right panel).

Supplemental Figure 5

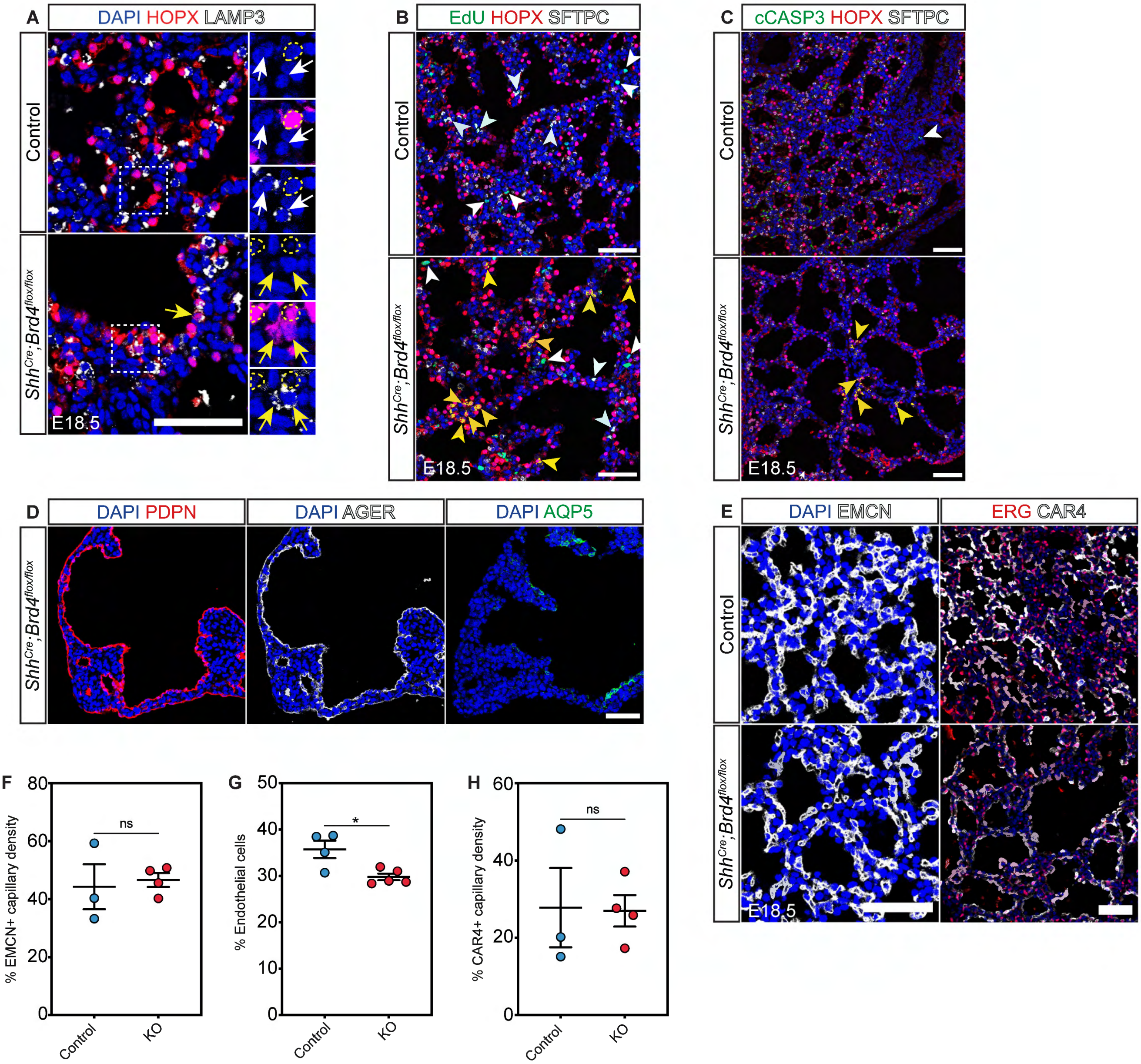

Supplementary Figure S5. BRD4 mutant lungs exhibit alveolar defects while maintaining vascular density. (A) IHC for HOPX and LAMP3 in control and mutant E18.5 lungs. Boxed areas represent magnified images presented to the right of each large panel. Dashed yellow circles mark AT1 cells, white arrows mark AT2 cells, and yellow arrows mark HOPX+ LAMP3+ cells (scale bars: 50 μm). (B) IHC for SFTPC, HOPX, and staining for EdU at E18.5. White arrowheads point at EdU+ cells. Yellow arrowheads point at HOPX+ SFTPC+ cells. (C) IHC for SFTPC, HOPX, and cleaved caspase 3 (cCASP3) in control and mutant E18.5 lungs (scale bars: 50 μm). White arrowheads point at cCASP3+ cells. Yellow arrowheads point at HOPX+ SFTPC+ cells. (D) IHC for PDPN, AGER, and AQP5 in mutant E18.5 lungs focusing on the distal cystic airway structures (scale bar: 50 μm). (E) IHC for EMCN (left) and ERG and CAR4 (right) in control and mutant E18.5 lungs (scale bars: 50 μm). (F-H) Quantification of capillary density and endothelial cell number in control and mutant E18.5 lungs as shown in panel (E). Data are represented as mean ± SEM. Two-tailed t tests: ns: not significant, *$p \leq 0.05$, $n \geq 3$ for each group.

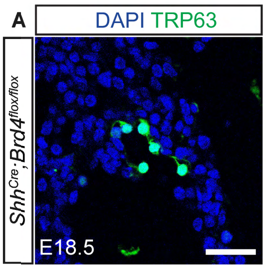

Supplementary Figure S6. Rare ectopic basal cells in the alveolar region in BRD4 mutants. (A) IHC for the basal cell marker, TRP63. Scale bar: 100 µm.

Supplemental Figure 7

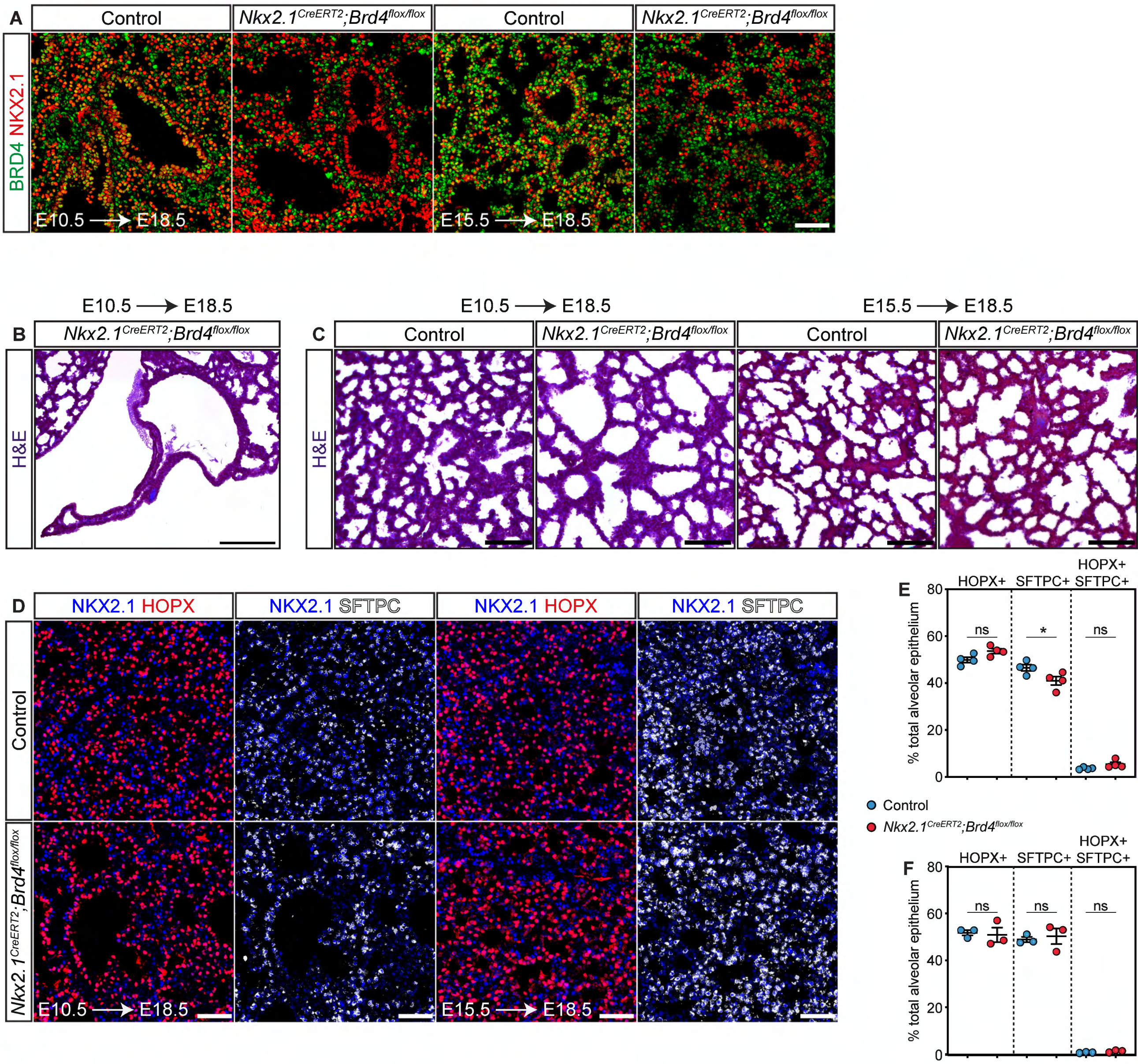

Supplementary Figure S7. **BRD4 governs temporal alveolar cell fate acquisition.** (A) IHC for BRD4 and NKX2.1 in control and mutant E18.5 lungs with (left) E10.5 tamoxifen induction and (right) E15.5 tamoxifen induction (scale bar: 50 µm). (B) Hematoxylin and eosin (H&E) staining of mutant lung at E18.5 with E10.5 tamoxifen induction focusing on the distal cystic airway structure (scale bar: 200 µm). (C) Hematoxylin and eosin (H&E) staining of mutant lung at E18.5 with (left) E10.5 tamoxifen induction and (right) E15.5 tamoxifen induction focusing on the distal alveolar regions (scale bar: 100 µm). (D) IHC for NKX2.1, HOPX, and SFTPC in control and mutant E18.5 lungs with (left) E10.5 tamoxifen induction and (right) E15.5 tamoxifen induction (scale bar: 50 µm). (E-F) Quantification of AT1, AT2, and HOPX+ SFTPC+ cells at E18.5 in control versus mutant lungs with (E) E10.5 tamoxifen induction and (F) E15.5 tamoxifen induction as shown in panel (D). Quantification data are represented as mean ± SEM. Two-tailed t tests: ns: not significant, $^*p \leq 0.05$, $n \geq 3$ for each group.

Supplemental Figure 8

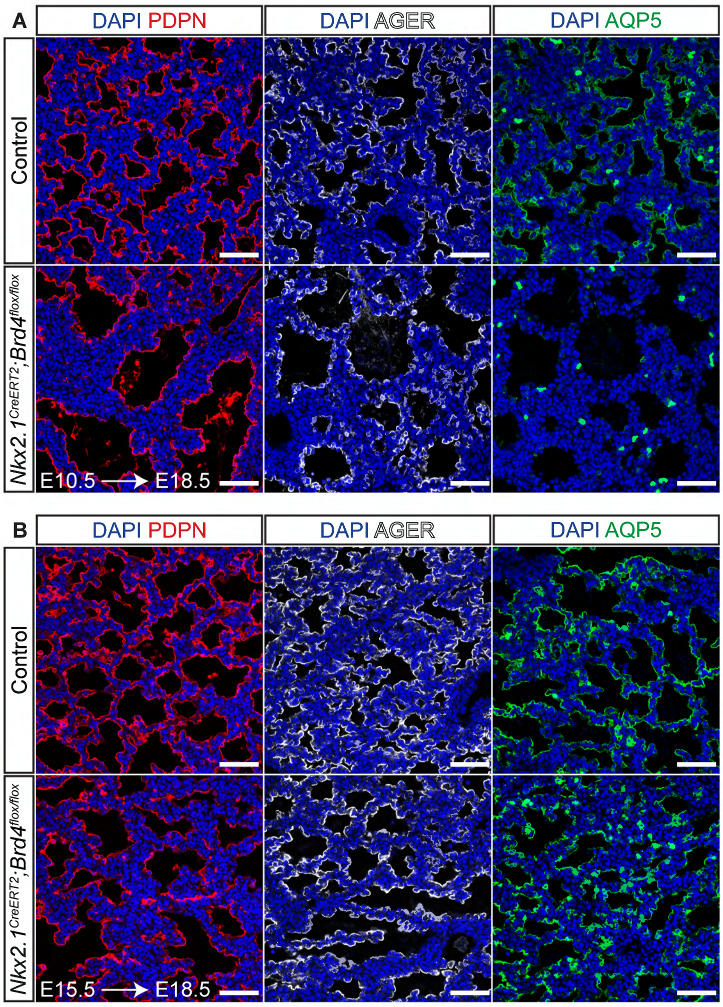

Supplementary Figure S8. BRD4 regulates AT1 maturation only early in lung development. (A and B) IHC for PDPN, AGER, and AQP5 in control and Nkx2.1^{CreERT2};Brd4$^{flox/-flox}$ mutant E18.5 lungs when deleted at E10.5 (A) and E15.5 (B) (scale bar: 50 μm).

Supplemental Figure 9

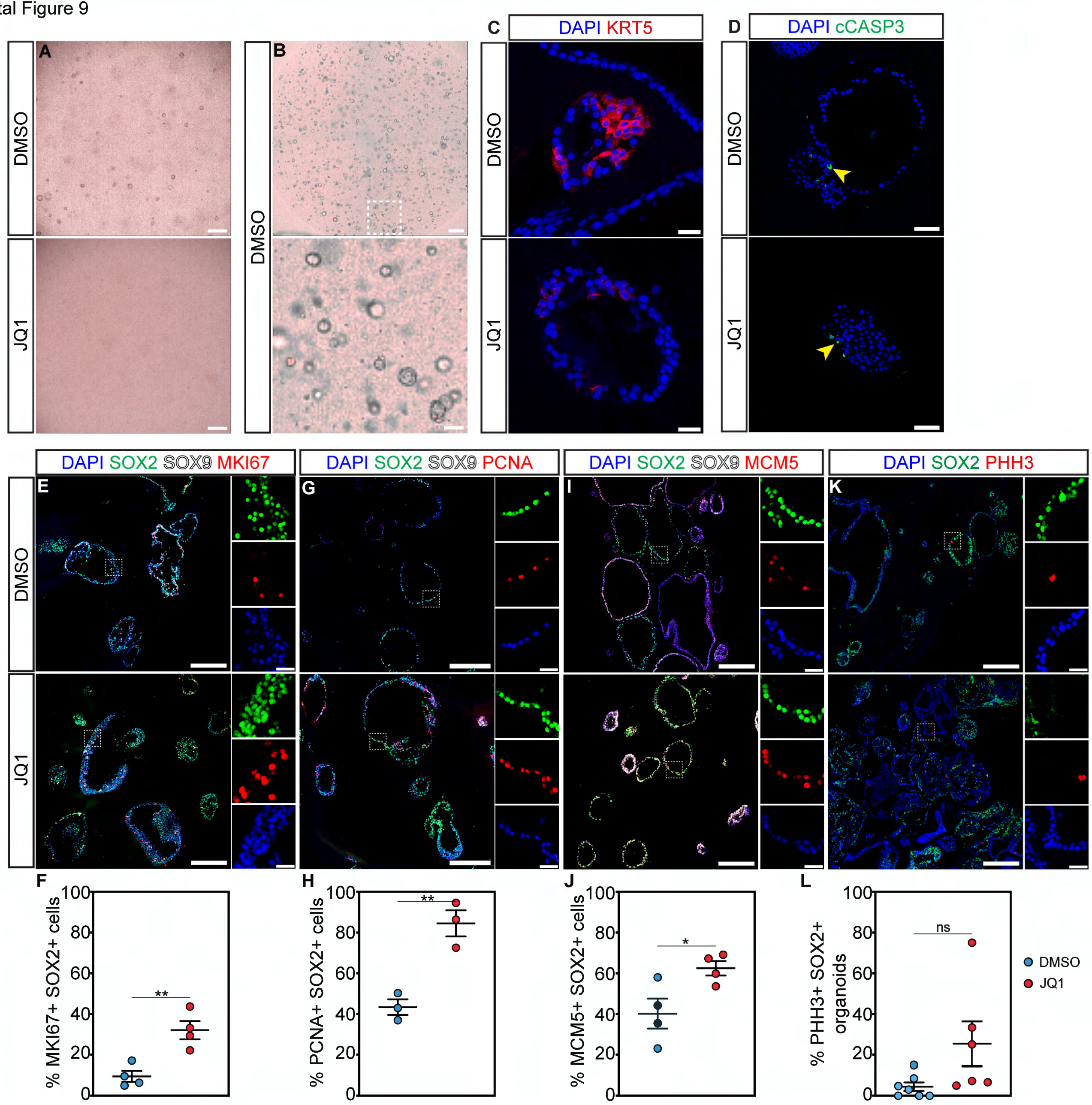

Supplementary Figure S9. BRD4 bromodomains inhibition causes differentiation and proliferation changes in hESC-derived lung organoids. (A) Overlapped brightfield and fluorescent images of hESC-derived airway organoids with *SCGB3A2*^{mCherry} reporter on day 10 after DMSO (top) and JQ1 (bottom) treatment at day 0 (scale bar: 500 µm). (B) Overlapped brightfield and fluorescent images of DMSO treated hESC-derived airway organoids with *SCGB3A2*^{mCherry} reporter on day 10 (scale bar: 1 mm). Boxed area marks the magnified region of organoids, as shown in the bottom panel (scale bar: 200 µm). (C) IHC for KRT5 in DMSO and JQ1 treated organoids (scale bar: 50 µm). (D) IHC for cleaved caspase 3 (cCASP3) in DMSO and JQ1 treated organoids (scale bar: 50 µm). Yellow arrowheads point to cCASP3+ cells. (E-L) Quantification and IHC for (E) SOX2, SOX9, and MKI67, (G) SOX2, SOX9, and PCNA, (I) SOX2, SOX9, and MCM5, and (K) SOX2, PHH3 in DMSO and JQ1 treated organoids (scale bar: 200 µm). Dashed white boxes mark magnified areas of SOX2+, MKI67+ cells (E), SOX2+, PCNA+ cells (G), SOX2+, MCM5+ cells (I), and PHH3+ cells (K) (scale bar: 50 µm). Quantification of percentages of (F) MKI67+, SOX2+ cells, (H) PCNA+, SOX2+ cells, (J) MCM5+, SOX2+ cells, and (L) PHH3+, SOX2+ organoids as shown in panel (E), (G), (I), and (K), respectively. Quantification data are represented as mean ± SEM. Two-tailed t tests: ns: not significant, *p ≤ 0.05, **p ≤ 0.01, n ≥ 3 for each group.
